# An unexpected role for Dicer as a reader of the unacetylated DNA binding domain of p53 in transcriptional regulation

**DOI:** 10.1126/sciadv.abi6684

**Published:** 2021-10-27

**Authors:** Xin Yang, Xingwu Wang, Zhiming Li, Shoufu Duan, Huan Li, Jian Jin, Zhiguo Zhang, Wei Gu

**Affiliations:** 1 Institute for Cancer Genetics, and Herbert Irving Comprehensive Cancer Center, Vagelos College of Physicians and Surgeons, Columbia University, 1130 Nicholas Ave., New York, NY 10032, USA.; 2 Mount Sinai Center for Therapeutics Discovery, Departments of Pharmacological Sciences and Oncological Sciences, Tisch Cancer Institute, Icahn School of Medicine at Mount Sinai, New York, NY 10029, USA.; 3 Department of Pediatrics and Department of Genetics and Development, Vagelos College of Physicians and Surgeons, Columbia University, 1130 Nicholas Ave., New York, NY 10032, USA.; 4 Department of Pathology and Cell Biology, Vagelos College of Physicians and Surgeons, Columbia University, 1130 Nicholas Ave., New York, NY 10032, USA.

## Abstract

Here, we identified Dicer as a major cellular factor that recognizes the DNA binding domain (DBD) of p53 in a manner dependent on its acetylation status. Upon binding the unacetylated DBD, Dicer is recruited to the promoters of p53 target genes, where it represses p53-mediated transcriptional activation. Conversely, knockdown or knockout of endogenous Dicer leads to up-regulation of p53-mediated transcriptional activation without increasing its protein levels. Moreover, Dicer-mediated repression is independent of its intrinsic endoribonuclease activity; instead, Dicer directly represses transcription by recruiting the SUV39H1 histone methyltransferase. However, upon DNA damage, Dicer-mediated repression is abrogated by stress-induced acetylation at the DBD of p53. Notably, the inability of acetylation-defective p53-3KR in transcription is partially but significantly restored upon loss of Dicer expression. Our study reveals that Dicer acts as an unexpected acetylation “reader” for p53 and thus has important implications regarding the mechanism of acetylation-mediated regulation of p53 transcriptional program.

## Teaser

Dicer acts as an unexpected cofactor in modulating promoter-specific regulation of p53 targets in an acetylation-dependent manner.

## INTRODUCTION

Although acetylation was initially identified as a posttranslational modification of histones (*1*, *2*), lysine acetylation is now recognized as a general protein modification that regulates multitude cellular functions in a manner analogous to protein phosphorylation in terms of its prevalence and biological significance in human diseases (*3*, *4*). Reversible protein acetylation and its modifying enzymes have been implicated in many cellular functions including transcription, DNA repair, and metabolism (*5*–*7*). p53 was one of the first nonhistone proteins shown to be functionally regulated by acetylation and deacetylation (*8*, *9*), and subsequent studies have established that p53 acetylation is critical for transcriptional regulation of p53 targets during stress responses (*10*–*12*). Moreover, recent studies showed that acetylation of specific lysine residues within the DNA binding domain (DBD) plays a critical role in promoter-specific regulation of p53 targets. For example, human p53 is acetylated at K120 (or K117 for mouse p53) by Histone acetyltransferase KAT5/8 (Tip60/MOF) (*13*, *14*), whereas K164 of human p53 (or K161 and K162 for mouse p53) is acetylated by CREB-binding protein (CBP) and p300 (*15*). These acetylation sites are mutated in human tumors and well conserved in all species known to encode p53. By generating acetylation-defective p53 knockin mice bearing lysine to arginine mutations (*p53-3KR*; K117R+K161R+K162R) at these acetylation sites, we found that p53-3KR loses its ability to activate specific cellular targets such as p21 and p53 upregulated modulator of apoptosis (PUMA) critical for cell cycle arrest, apoptosis, and senescence, but its ability to induce Murine double minute 2 (Mdm2) expression remains intact (*16*). These findings underscore the importance of acetylation in differentially modulating p53 responses and also suggest that these acetylation sites may act as a docking site for specific regulators of p53. Nevertheless, the identity of the regulators and precise mechanisms of acetylation-mediated action remain largely unknown.

## RESULTS

### Dicer interacts with p53 both in vitro and in vivo

**Fig. 1.**
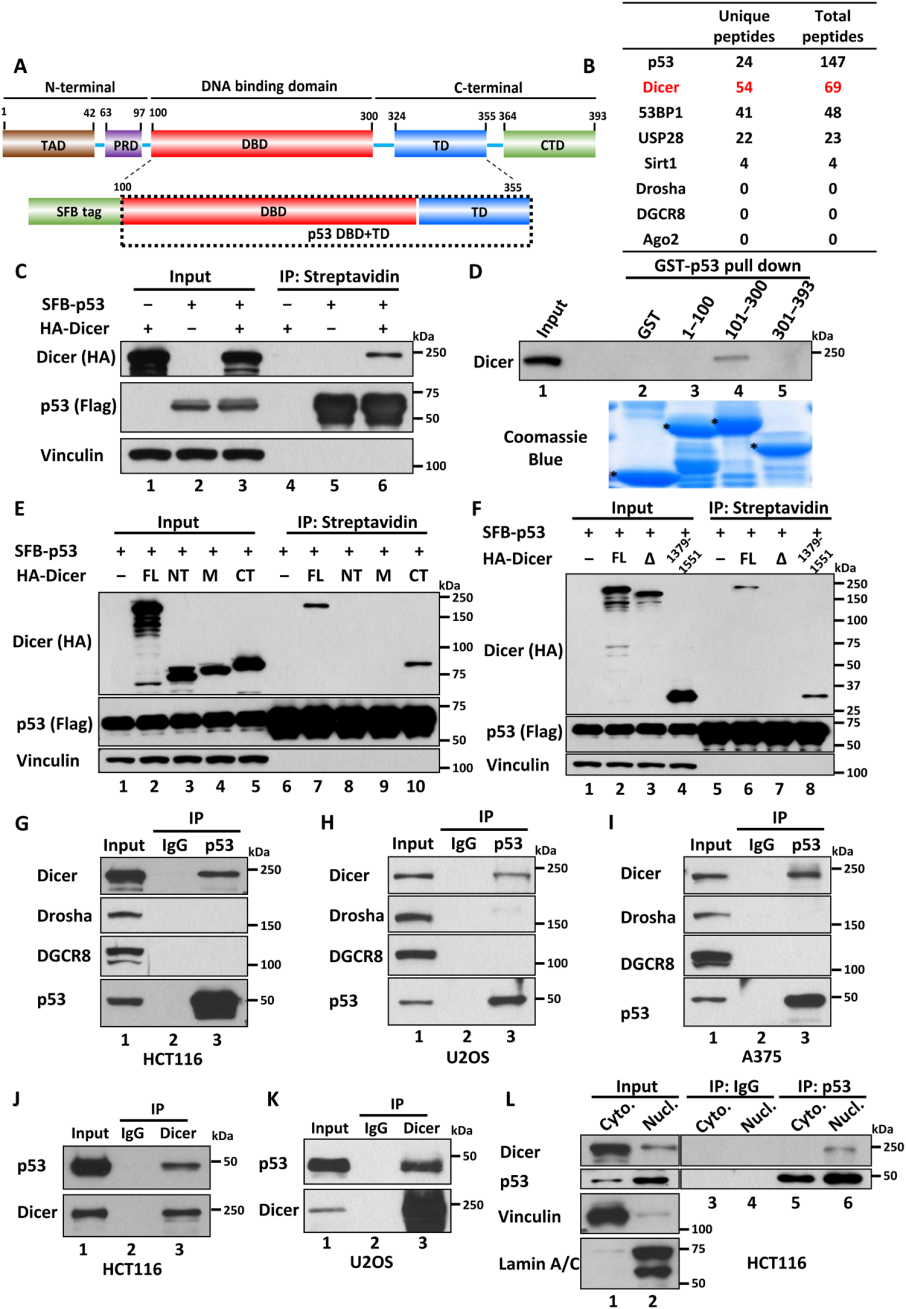
Dicer interacts with p53 both in vitro and in vivo. (**A**) Schematic diagram of the p53 domain. TAD, transactivation domain; PRD, proline domain; TD, tetramerization domain; CTD, C-terminal domain. (**B**) Count of peptide of p53 interacted protein was shown as a table. (**C**) Western blot analysis of the interaction between overexpressed p53 and Dicer in H1299 cells. IP, immunoprecipitation. (**D**) In vitro binding assay of GST-p53 amino acids 1 to 100, amino acids 101 to 300, or amino acids 301 to 393 and purified Dicer. (**E** and **F**) Western blot analysis of p53 and Dicer domains for their interaction in H1299 cells. FL, full length, amino acids 1 to 1922; NT, N-terminal, amino acids 1 to 626; M, middle, amino acids 627 to 1270; CT, C-terminal, amino acids 1271 to 1922; ∆, full-length Dicer lacking, amino acids 1379 to 1551. (**G** to **I**) Western blot analysis of endogenous interaction among p53, Dicer, Drosha, and DGCR8 in HCT116 (G), U2OS (H), or A375 (I) cells. (**J** and **K**) Western blot analysis of endogenous interaction between Dicer and p53 in HCT116 (J) or U2OS (K) cells. (**L**) Western blot analysis of the interaction between p53 and Dicer in the cytoplasm (Cyto.) or nucleus (Nucl.) fraction of HCT116 cells upon MG132 treatment. All data are shown as representative of three experiments.

p53 is regulated by an exquisite network of fine-tuning mechanisms that ensure proper responses to the various stress signals encountered by cells (*11*, *17*–*19*). To elucidate the molecular basis by which these acetylation events control promoter-specific activation of p53 targets, we tried to identify the major cellular factors that specifically interact with the DBD of p53 in human cells. To this end, cell extracts from a p53-null H1299 lung carcinoma cell line that stably expressed a human p53 fragment [SFB-p53 (amino acids 100 to 355)] with a N-terminal triple-epitope tag, SFB (S protein, FLAG tag, and streptavidin-binding peptide) (Fig. 1A) were subjected to multistep affinity chromatography (streptavidin–agarose beads and S protein–agarose beads) as previously described (*20*). The affinity-purified SFB-p53–associated proteins were analyzed by liquid chromatography–tandem mass spectrometry (LC-MS/MS). As expected, we identified several cellular proteins that are already known to interact with the DBD of p53 including Tumor suppressor p53-binding protein 1 (53BP1), Ubiquitin carboxyl-terminal hydrolase 28 (USP28), and Sirtuin 1 (Sirt1) from the complexes (*21*, *22*). Unexpectedly, MS revealed that 54 peptides matched with a protein called Dicer as a major component from the DBD-associated protein complexes (Fig.1B and fig. S1, A and B). Dicer is an endoribonuclease, which plays a central role in microRNA (miRNA)–mediated silencing and several other RNA interference (RNAi) pathways critically for posttranscriptional gene regulation (*23*–*27*). However, we failed to identify any peptide matched with other miRNA/RNAi processing proteins such as Drosha, DiGeorge syndrome critical region 8 (DGCR8), and Protein argonaute-2 (Ago2) (Fig. 1B and fig. S1B), suggesting that p53 specifically interacts with Dicer alone but not with RNA processing complexes.

Next, we examined the interactions between Dicer and p53 both in vitro and in vivo. To this end, we first cotransfected native H1299 cells with a SFB-tagged full-length p53 expression vector and hemagglutinin (HA)-tagged Dicer expression vector. Western blot (WB) analysis revealed that Dicer was readily detected in the immunoprecipitated complexes of SFB-p53 (Fig. 1C). Moreover, by using glutathione *S*-transferase (GST) pull-down assays, we validated that the DBD of p53 (amino acids 101 to 300) alone is responsible for the direct interaction with Dicer (Fig. 1D and fig. S1C). To further characterize the interaction between Dicer and p53 in human cells, we tested the binding affinity between p53 and different domains of Dicer (N-terminal domain, amino acids 1 to 626; M, middle domain, amino acids 627 to 1270; C-terminal domain (CTD), amino acids 1271 to 1922) (fig. S2A). As shown in Fig. 1E, p53 was showed to interact with the CTD of Dicer. Further subdomain mapping showed that a small region (amino acids 1379 to 1551) within the CTD was sufficient for interacting with p53, whereas the Dicer mutant (Dicer-Δ) lacking this region completely lost its ability to interact with p53 (Fig. 1F and fig. S2B).

To evaluate this interaction under more physiological settings, we performed the co-immunoprecipitation (co-IP) assay in human colorectal cancer HCT116 cells expressing wild-type p53. As shown in Fig. 1G, endogenous Dicer, but not Drosha or DGCR8, was coimmunoprecipitated with endogenous p53 by an anti-p53–specific monoclonal antibody (DO-1) but not by the anti–immunoglobulin G (IgG) control. Similar results were also obtained in human osteosarcoma U2OS cells (Fig. 1H) and human melanoma A375 cells (Fig. 1I), both of which express wild-type p53. Conversely, endogenous wild-type p53 was coprecipitated with endogenous Dicer by an anti-Dicer–specific polyclonal antibody but not by the anti-IgG control (Fig. 1, J and K). To further validate the specific interaction between p53 and Dicer, we performed the co-IP assays in p53-null HCT116 cells. As expected, Dicer was not detected in the immunoprecipitated complexes by the same p53 antibody in p53-null HCT116 cells (fig. S3A). Furthermore, the Dicer-p53 interaction was also detected in human cancer cell lines expressing mutant p53 (fig. S3, B to D). Although endogenous Dicer is mainly localized in the cytoplasm for its RNA processing role, a significant proportion of Dicer is also present in the nucleus with the functions not well defined (*28*). Notably, Western blot analysis revealed that Dicer was only detectable in the immunoprecipitated complexes of p53 from the nucleus of HCT116 cells (Fig. 1L). Similar results were also confirmed in U2OS cells (fig. S3E). Together, these data demonstrate that Dicer is a bona fide binding partner of p53 and also suggest that this interaction is potentially important for Dicer function independent of its RNA process activities.

### Dicer acts as a transcriptional repressor of p53

**Fig. 2.**
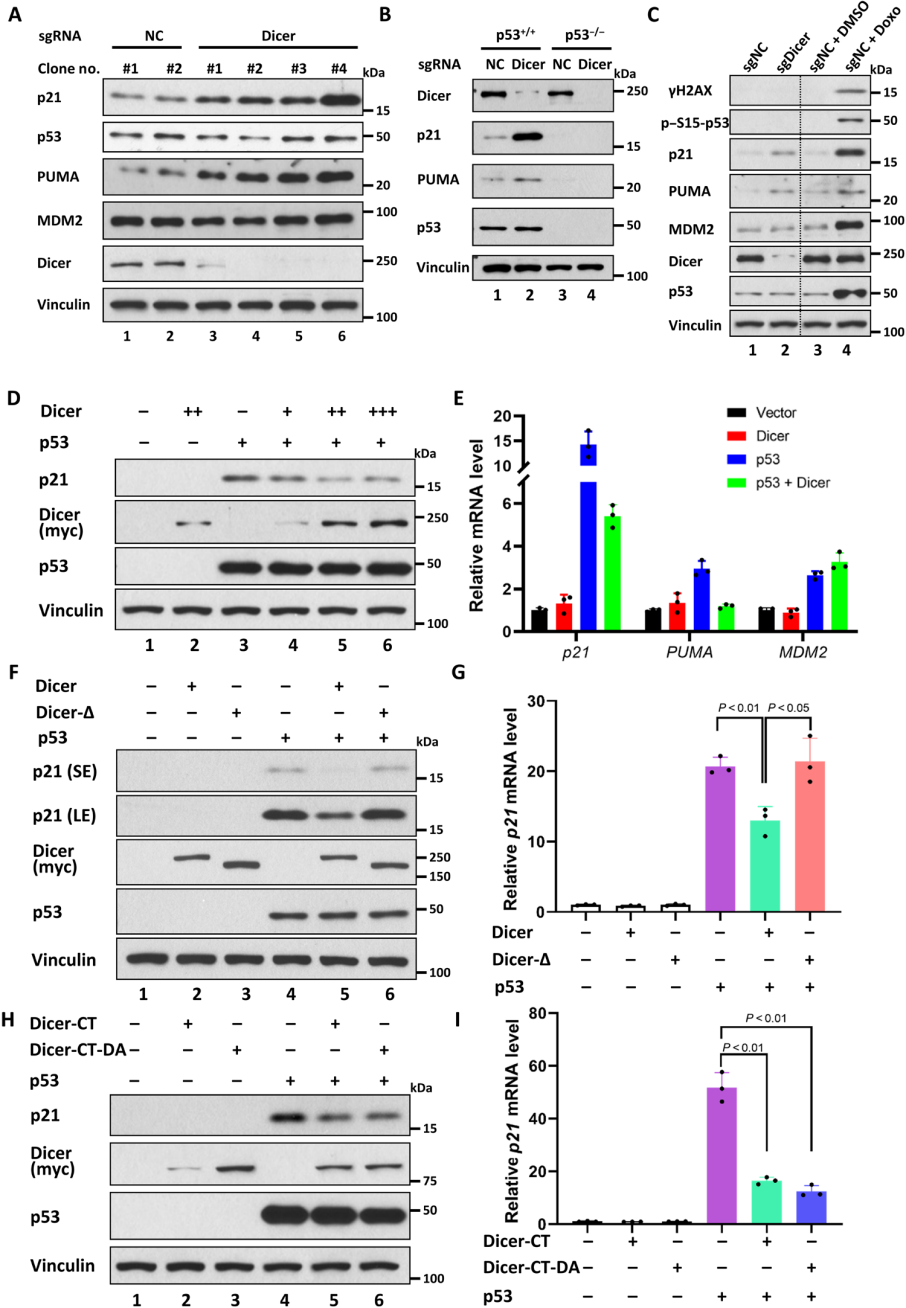
Dicer acts as a transcriptional repressor of p53. (**A**) Western blot analysis of the effect of different Dicer CRISPR-Cas9 knockout clones on p53 activity in HCT116 cells. sgRNA, single-guide RNA. (**B**) Western blot analysis of the effect of Dicer knockout in p53^+/+^ or p53^−/−^ HCT116 cells. (**C**) Western blot analysis of the DNA damage response effect of Dicer knockout in p53 wild-type HCT116 cells. CRISPR-Cas9 negative control cells treated with doxorubicin (0.2 μg/ml) are positive control in DNA damage response. DMSO, dimethyl sulfoxide. sgNC, sgRNA Negative Control. (**D**) Western blot analysis of H1299 cells transfected with p53 or cotransfected with p53 and increasing amount of Dicer. (**E**) Quantitative polymerase chain reaction (qPCR) assay of the *p21*, *PUMA*, and *Mdm2* genes regulated by the p53 and/or Dicer transfection in H1299 cells. (**F** and **G**) Western blot analysis and qPCR assay for detecting p21 level in H1299 cells transfected with p53 or cotransfected with p53 and Dicer or Dicer-∆. (**H** and **I**) Western blot analysis and qPCR assay for detecting p21 level in H1299 cells transfected with p53 or cotransfected with p53 and Dicer CT or CT-D1320A. Error bars in qPCR assay indicate means ± SD. *n* = 3 technical replicates. All data are shown as representative of three experiments.

Above data indicate that Dicer interacts with p53 in the nucleus. Thus, to evaluate the functional consequences of the p53-Dicer interaction, we first examined whether inactivation of endogenous Dicer influences the activities of p53 in human cancer cells. CRISPR-mediated inactivation of Dicer markedly elevated the expression of p53 targets, such as p21 and PUMA, without affecting the steady-state levels of endogenous p53 in HCT116 colorectal carcinoma cells (lane 3, Fig. 2A; fig. S4A). Similar results were also obtained in other independent Dicer knockout HCT116 clones (lanes 4 to 6, Fig. 2A) or human lung carcinoma A549 cells upon Dicer knockdown (fig. S4B). Further analysis identified additional p53 targets that were up-regulated upon inactivation of Dicer, including the genes that are critically involved in p53-mediated cell cycle arrest and apoptosis (fig. S4, C and D). However, knockdown of other components of the miRNA/small interfering RNA (siRNA) processing complexes, such as Drosha or DGCR8, had no obvious effect on p53-mediated transactivation (fig. S4E). The induction of p21 and PUMA expression upon Dicer knockout was completely abrogated in isogenic HCT116 p53^−/−^ cells (Fig. 2B), indicating that Dicer-mediated effects are p53 dependent. Because loss of Dicer only induced modest activation of p53 targets such as p21 and PUMA under unstressed conditions (Fig. 2C and fig. S4F), these data also suggest that other cellular factors are also required for the robust activation of p53 function during stress responses. Moreover, we failed to detect any obvious up-regulation of either γH2AX levels or Ser^15^ p53 phosphorylation levels, two well-known markers for DNA damage (Fig. 2C), suggesting that the observed activation of p53 function upon loss of Dicer expression is not caused by indirect effects from DNA damage responses. Consistent with this notion, the levels of p53 protein remained unchanged upon Dicer knockdown (Fig. 2A).

Next, we examined whether Dicer directly regulates p53-dependent transcriptional activity. To this end, p53-null H1299 cells were cotransfected with expression vectors encoding p53 alone, Dicer alone, or p53 and Dicer together. As expected, p53 expression induced transactivation of p21 expression (lane 3, Fig. 2D; Fig. 2E). However, p53-dependent transactivation of p21 was strongly repressed upon coexpression of Dicer in a dosage-dependent manner without affecting the p53 levels (lanes 4 to 6, Fig. 2D). Moreover, the ability of Dicer to repress p53-mediated p21 transactivation was lost upon deletion of the small region at the CTD (Dicer-Δ, lane 6 versus lane 5, Fig. 2F; Fig. 2G and fig. S4G), which abrogates its interaction with p53. The CTD (amino acids 1271 to 1922), which is responsible for the interaction with p53, fully retained its ability to suppress p53-mediated transactivation (lane 5 versus lane 4, Fig. 2H; Fig. 2I). Notably, Dicer-mediated repression was also retained by Dicer-D1320A, an endoribonuclease-deficient point mutant (lane 6 versus lane 5, Fig. 2H; Fig. 2I) (*29*). Thus, these data demonstrate that p53-Dicer interaction is essential for its suppression of p53-dependnet transcriptional activation and also suggest that Dicer-mediated repression is independent of its intrinsic endoribonuclease activity. Both Dicer knockdown and Dicer overexpression significantly modulated p53-dependent activation of p21 and PUMA but had no obvious effect on Mdm2 or Tigar expression (Fig. 2, A, C, and E, and fig. S4, A and C). Together, these data demonstrate that the Dicer-p53 interaction is critical for regulating p53-mediated transactivation in a promoter-specific manner.

### Regulation of the dicer-p53 interaction by acetylation

**Fig. 3.**
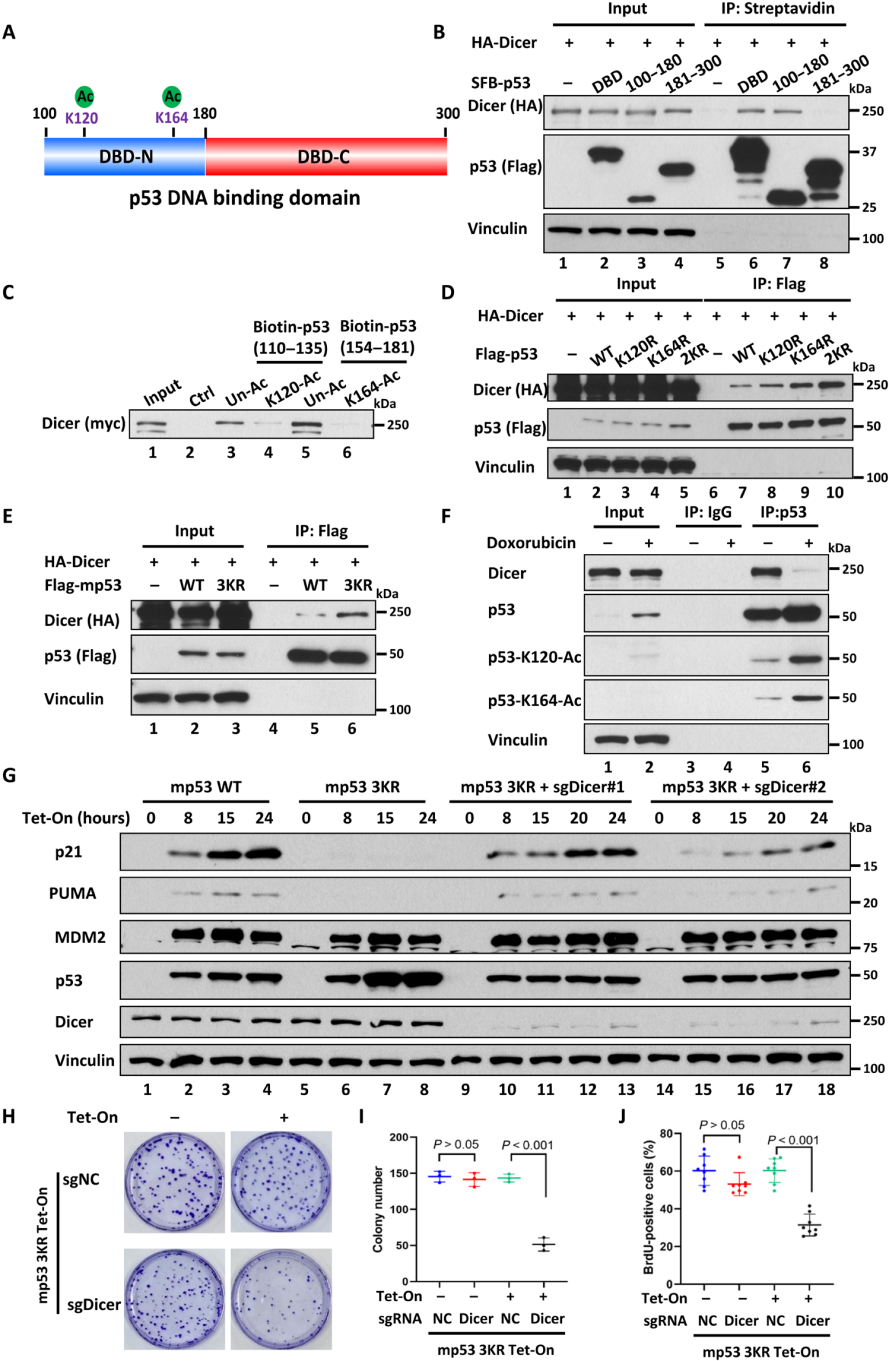
Regulation of the Dicer-p53 interaction by acetylation. (**A**) Schematic diagram of the p53 DBD. (**B**) Western blot analysis of Dicer and p53 DBD subdomains (DBD-N, amino acids 100 to 180; and DBD-C, amino acids 181 to 300) for their interaction in H1299 cells. (**C**) In vitro binding assay of biotin-conjugated K120 and K164 unacetylated or acetylated p53 peptides and Dicer. (**D**) Western blot analysis of Dicer and human p53 wild-type (WT), K120R, K164R, and 2KR (120/164) for their interaction in H1299 cells. (**E**) Western blot analysis of Dicer and mouse p53 WT and 3KR (117/161/162) for their interaction in H1299 cells. (**F**) Western blot analysis of the endogenous interaction between p53 and Dicer upon MG132 plus TSA/NAM treatment or doxorubicin (0.2 μg/ml) plus TSA/NAM treatment of HCT116 cells. (**G**) Western blot analysis of p53 activity in H1299 mouse p53 WT, 3KR, or 3KR Dicer knockdown Tet-On cells with or without induction in time dependent manner. (**H** and **I**) Representative image and quantitative colony formation number in H1299 mouse p53 3KR Tet-On sgNC or sgDicer cells with or without doxycycline (0.1 μg/ml) for 14 days. *n* = 3 for biological replicates. (**J**) Quantitative BrdU-positive ratio in H1299 mouse p53 3KR Tet-On sgNC or sgDicer cells with or without doxycycline (0.1 μg/ml) for 24 hours. *n* = 8 biological replicable images. Error bars indicate means ± SD. All data are shown as representative of three experiments.

Our previous studies showed that acetylation of the DBD at K120 and K164 is critical for p53-mediated activation of p21 and PUMA but has no obvious effect on Mdm2 or Tigar expression (*16*), in the manner reminiscent of Dicer-mediated effects on these different p53 target promoters. To elucidate whether the Dicer-p53 interaction is modulated by acetylation, we first identified a specific region within the DBD of p53 responsible for interacting with Dicer (Fig. 3A). As indicated in Fig. 3B, the DBD-N (amino acids 100 to 180) was showed to strongly interact with Dicer, whereas no obvious binding was detected between the DBD-C (amino acids 181 to 300) and Dicer, suggesting that the region between amino acids 100 and 180 is the docking site for Dicer binding. To examine that the Dicer-p53 interaction is regulated by acetylation, we performed an in vitro binding assay of the purified recombinant Dicer protein with unacetylated versus acetylated peptides of p53 (fig. S5A). As shown in Fig. 3C, Dicer interacted with the unacetylated [Biotin-p53–(amino acids 110 to 135)–Un-Ac] but not the K120 acetylated [Biotin-p53–(amino acids 110 to 135)–K120-Ac] peptide (lane 4 versus lane 3), while the similar results were also obtained when unacetylated or acetylated K164 peptide [Biotin-p53–(amino acids 154 to 181)–Un-Ac versus Biotin-p53–(amino acids 154 to 181)–K164-Ac] was used in the same assays. Because we have identified the small region (amino acids 1379 to 1551) within the CTD of Dicer as the docking site for p53 binding, we also examined the interaction of this docking site with unacetylated versus acetylated peptides of p53. Both K120 acetylated and K164 acetylated peptides were able to abrogate their bindings with this region derived from Dicer protein (fig. S5B). These results demonstrate that both K120 acetylation and K164 acetylation of p53 are critically involved in modulating the interaction between p53 and Dicer.

Next, to determine whether p53 acetylation at K120 and K164 is critical for its interaction with Dicer in human cells, we tested whether the p53-Dicer interaction is affected by different acetylation-deficient mutants of p53: p53-K120R (K➔R mutation at K120), p53-K164R (K➔R mutation at K164), or p53-2KR (K➔R mutation at K120 and K164). Consistent with the in vitro binding data shown above, the interaction between p53 and Dicer in human cells was enhanced when acetylation-deficient mutants were expressed (lanes 8 to 10 versus lane 7, Fig. 3D). Similar results were also obtained when Dicer was coexpressed with an acetylation-deficient mouse p53 mutant at the equivalent sites mp53-3KR (K➔R mutation at K117, K161, and K162) (lane 6 versus lane 5, Fig. 3E), suggesting that acetylation-mediated regulation of the Dicer-p53 interaction is well conserved in mouse cells. Moreover, because the levels of p53 acetylation at K120 and K164 are induced by DNA damage, we examined the effects of genotoxic stress on the p53-Dicer interaction. As expected, the levels of p53 acetylation at K120 and K164 were markedly induced by the treatment of doxorubicin, a topoisomerase inhibitor (lane 2 versus lane1, Fig. 3F). Notably, despite increased levels of p53 protein, Dicer polypeptides were barely detectable in the immunoprecipitated complexes with acetylated p53 from doxorubicin-treated cells (lane 6 versus lane 5, Fig. 3F). Similar results were also obtained in human osteosarcoma U2OS cells (fig. S5C). Together, these results demonstrate that Dicer preferentially binds to unacetylated forms of p53, but the Dicer-p53 interaction is suppressed by p53 acetylation.

By using acetylation-defective p53 mutant knockin mouse models, we previously showed that an acetylation-deficient mouse p53 mutant (mp53-3KR) losses its ability to activate p53-target genes such as p21 and PUMA upon DNA damage (*16*). Because above data indicate that acetylation-mediated suppression of the Dicer-p53 interaction plays a critical role for p53 activation in response to DNA damage, it is very likely that the failure of mp53-3KR in transcription activation may be caused by inability of blocking its interaction with Dicer induced by acetylation. To this end, we first generated the mp53-3KR Tet-On inducible cell line in which mp53-3KR expression can be induced by tetracycline and then tested whether the inability of mp53-3KR in transcriptional activation can be reversed by knockdown of Dicer expression. Consistent with our previous study, mp53-3KR was able to activate expression of Mdm2, but not p21 or PUMA (lanes 5 to 8 versus lanes 1 to 4, Fig. 3G). Notably, upon knockdown of Dicer expression by Dicer-specific single-guide RNA (sgRNA), activation of p21 and PUMA expression was partially restored although the levels of Mdm2 expression remained the same (lanes 9 to 13 and lanes 14 to 18, Fig. 3G). To exclude the possibility that p53 activation induced by loss of Dicer expression is caused by indirect effects from miRNA/siRNA processing, we test whether the inability of mp53-3KR in transcriptional activation can also be reversed by other components of miRNA/siRNA processing complexes. To this end, CRISPR-mediated knockdown of Drosha or DGCR8 was also performed in the same mp53-3KR Tet-On inducible cell line. However, in contrast to Dicer knockdown, no obvious activation of p21 or PUMA expression was detected in Drosha or DGCR8 knockdown cells (lanes 4 to 6 versus lanes 7 to 14, fig. S6A), validating the direct effect of p53 function induced by Dicer. To corroborate with these findings, we performed the similar experiments by using p53^3KR/3KR^ mouse embryonic fibroblasts (MEFs) derived from acetylation-defective p53-3KR knockin mice. As shown in fig. S6B, upon knockdown of Dicer in p53^3KR/3KR^ MEFs by sgRNA, the functionality of p53-3KR in transcriptional activation of p21and PUMA was partially restored, whereas the levels of Mdm2 expression remained the same (lanes 2, 4, and 6). This result demonstrates that the failure of the acetylation-deficient p53 mutant in transcriptional activation at specific targets can rescued by knockdown of Dicer expression in vivo.

Moreover, by using both cell growth colony assays and 5-bromo-2′-deoxyuridine (BrdU) staining, we further validated that the ability of p53-3KR in cell growth repression was also restored upon Dicer knockdown (Fig. 3, H to J). These data demonstrate that the failure of the acetylation-deficient p53 mutant in transcriptional activation can be markedly rescued by knockdown of Dicer expression, underscoring the important role of acetylation in modulating Dicer-mediated repression of p53 transactivation in vivo.

### Mechanistic insights into dicer-mediated regulation of p53 functions

**Fig. 4.**
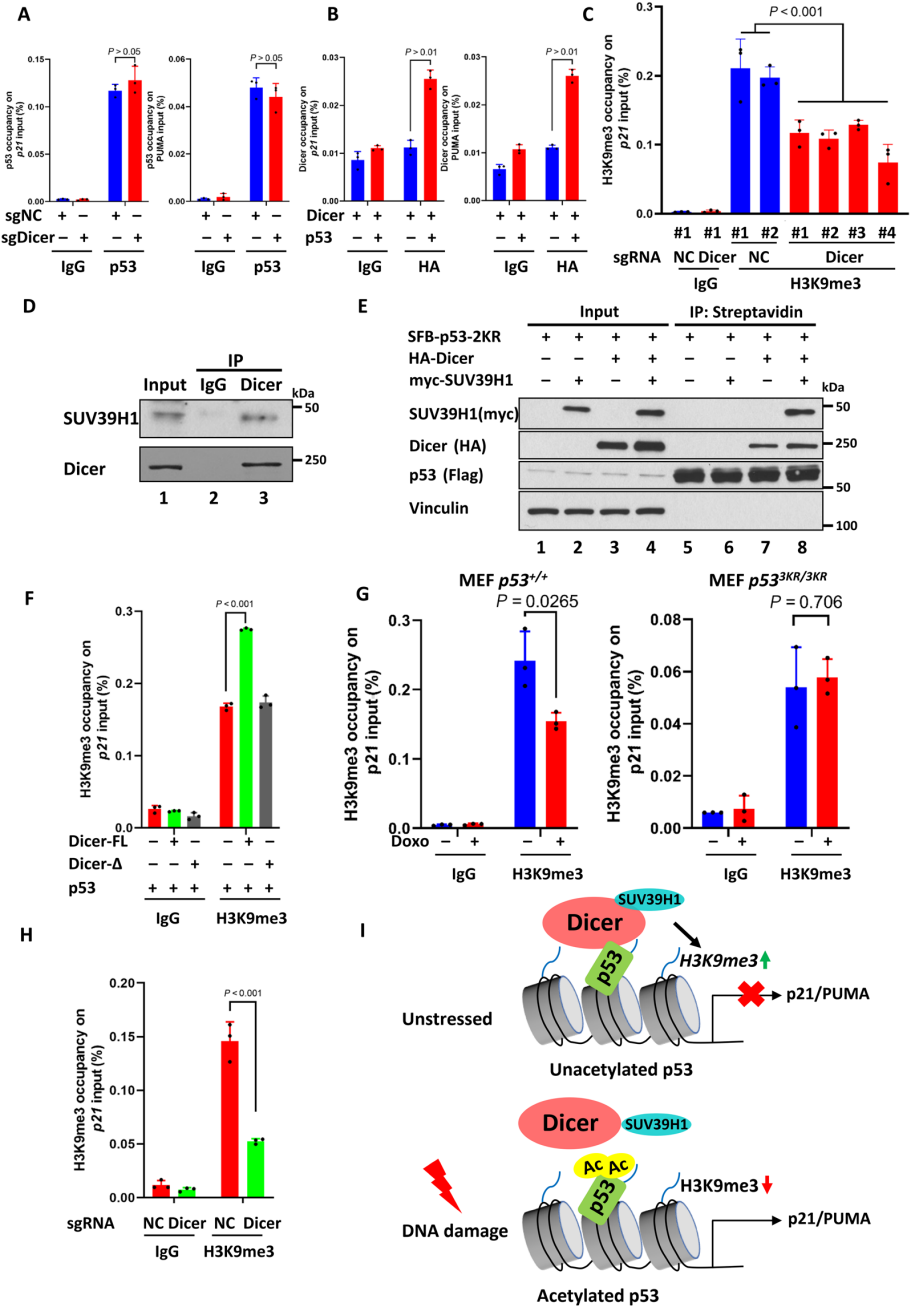
Mechanistic insights into Dicer-mediated regulation of p53 functions. (**A**) ChIP analysis of the Dicer overexpression mediated effect on p53 at the *p21* or *PUMA* promoter in HCT116 cells. (**B**) ChIP analysis of the p53 effect on Dicer at the *p21* or *PUMA* promoter in H1299 cells by transfected Dicer with or without p53. (**C**) ChIP analysis of the Dicer knockout mediated effect on H3K9me3 at the *p21* promoter in HCT116 cells. Two negative control clones and four Dicer knockout clones were examined. Significance was assessed in indicated comparisons by one-way analysis of variance (ANOVA). (**D**) Western blot analysis of endogenous interaction between Dicer and SUV39H1 in HCT116 cells. (**E**) Western blot analysis of the interaction among overexpressed p53, Dicer, and SUV39H1 in H1299 cells. (**F**) ChIP analysis of the overexpressed Dicer effect on H3K9me3 at the *p21* promoter in H1299 by transfected p53 plus Vector, Dicer, or Dicer-∆. (**G**) ChIP analysis of the H3K9me3 at the *p21* promoter in MEF *p53^+/+^* (left) or MEF *p53^3KR/3KR^* (right) cells with TSA/NAM or doxorubicin (0.2 μg/ml) plus TSA/NAM treatment. (**H**) ChIP analysis of the Dicer effect on H3K9me3 at the *p21* promoter in H1299 mouse p53 3KR Tet-On sgNC or sgDicer cells. (**I**) Work model of Dicer-mediated transcriptional regulation on p53. Error bars indicate means ± SD. *n* = 3 for technical replicates. All data are shown as representative of three experiments.

To elucidate the mechanism by which Dicer represses p53-dependent transcription, we first tested whether the DNA binding activity of p53 is inhibited by Dicer expression. As shown in Fig. 4A and fig. S7A, chromatin IP (ChIP) assays revealed that neither Dicer knockout nor overexpression had any obvious effect on the DNA binding activity of p53 on the promoters of p21 and PUMA, suggesting that the DNA binding is not the key factor in Dicer-mediated regulation. Next, we examined whether Dicer can be recruited to the target promoters of p53. Upon coexpressed with p53, Dicer was significantly recruited to the promoter of p21 and PUMA (Fig. 4B). Consistent with the binding data (Fig. 3E), the recruitment of Dicer was more effective when the acetylation defective mutant p53-3KR was expressed (fig. S7C). To evaluate the functional consequence of Dicer recruitment, we monitored the potential changes of a number of histone modification markers affected by Dicer knockout at the p21 and PUMA promoters, including H3K18 acetylation (H3K18Ac), H3K27 acetylation (H3K27Ac), H3K4 monomethylation (H3K4me1), H3K9 trimethylation (H3K9me3), and H3K27 trimethylation (H3K27me3). As shown in fig. S7D, the levels of H3K9me3 at the promoters of p21 and PUMA were markedly reduced upon Dicer knockout; in contrast, no change or very modest change was observed for the levels of other histone markers. Dicer knockout-induced reduction of H3K9me3 levels at the p21 and PUMA promoter was further confirmed in multiple independent clones of Dicer knockout cells (Fig. 4C and fig. S8A). Together, these data indicate that Dicer does not affect the DNA binding activity of p53 but can be recruited to the promoters of p53 target genes and subsequently affecting histone modification.

Notably, histone-lysine methyltransferase SUV39H1 is a key enzyme to regulate H3K9me3 levels (*30*). Although SUV39H1 cannot directly interact with p53, previous studies showed that SUV39H1 is critically involved in repressing p53-mediated transactivation (*31*). By using co-IP assays, we found that endogenous Dicer interacts with endogenous SUV39H1 in human cancer cells (Fig. 4D). By using in in vitro pull-down assays, we found that purified Dicer protein was able to bind a recombinant SUV39H1 (fig. S8B), indicating that SUV39H1 and Dicer interact directly. When p53 was coexpressed with SUV39H1 in human cells, SUV39H1 was undetectable in the immunoprecipitated complexes of p53 (lane 6, Fig. 4E). However, upon coexpression of Dicer in those cells, both Dicer and SUV39H1 were readily presented in the immunoprecipitated complexes of p53, suggesting that Dicer acts as a bridge for the p53-SUV39H1 interaction. The levels of SUV39H1 recruitment were significantly increased when both p53 and Dicer were also present at the promoters of p21 and PUMA; conversely, no significant increase was detected at the promoters of Mdm2 and Tigar under the same conditions (fig. S8C).

Next, we tested whether the p53-Dicer interaction is able to promote the H3K9me3 levels at the p53 target promoters. As shown in Fig. 4F and fig. S9A, the levels of H3K9me3 at the promoters of p21 and PUMA were significantly enhanced by coexpression of p53 with Dicer but not Dicer-Δ, a Dicer mutant defective for interacting with p53. Furthermore, because the p53-Dicer interaction can be suppressed by stress-induced acetylation, we tested whether the H3K9me3 levels at the p53 target promoters are regulated in response to DNA damage. As shown in Fig. 4G and fig. S9B, the levels of H3K9me3 at the promoters of p21 and PUMA were largely reduced upon DNA damage in wild-type MEFs. These results suggest that acetylation of p53 is crucial for blocking the Dicer-SUV39H1 recruitment for down-regulating H3K9me3 levels at p53 target promoters during stress responses. Last, because p53-3KR loses its ability to suppress its interaction with Dicer upon DNA damage, we tested whether the effects can be recapitulated by Dicer knockdown. The reduction of the H3K9me3 levels at the p53 target promoters was observed by Dicer knockdown in p53-3KR expressing cells (Fig. 4H and fig. S9C). These data demonstrate that Dicer represses p53-mediated transactivation by interacting with SUV39H1 and up-regulating H3K9me3 levels at the p53 target promoters.

## DISCUSSION

The Dicer ribonuclease modulates gene expression at the posttranscriptional level by processing miRNAs and siRNAs (*23*–*27*). Unexpectedly, we have found that Dicer also acts as a transcriptional regulator that binds the p53 tumor suppressor and down-regulates its transactivation activity. When cells experience various forms of stress, including DNA damage, p53 regulates the transcription of specific subsets of p53-target genes, which, in turn, elicit the appropriate cellular response, such as cell cycle arrest or apoptosis. In stressed cells, the transcriptional activity of p53 is tightly regulated by signaling pathways that acetylate the p53 protein. However, the molecular mechanisms by which acetylation activates p53-mediated transcription are not well understood. We showed that, in unstressed cells, Dicer interacts with the DBD of p53 and represses its transcriptional activity. Notably, however, upon DNA damage, Dicer-mediated repression is abrogated by stress-induced acetylation of the p53 DBD (Fig. 4I). Thus, by acting as a “reader” of the acetylation state of p53, Dicer is a bona fide transcriptional repressor that governs the expression of p53 target genes and prevents inappropriate activation of the p53 pathway in unstressed cells.

The role of Dicer in tumor development is complex. Although mutations in Dicer are commonly observed in many types of cancers, it is rare to find tumors with mutations on both copies of Dicer and that loss of heterozygosity resulting in complete deficiency of Dicer function is extremely rare (*32*). The studies from Dicer mutant mice indicate that Dicer functions as a haploinsufficient tumor suppressor in vivo (*33*). However, while loss of a single allele of Dicer enhanced tumorigenesis, loss of both copies of Dicer led to p53 activation and inhibition of tumorigenesis (*34*).The molecular mechanism by which its complete loss promotes p53 activation is not completely understood. Dicer is implicated in processing the DNA damage response noncoding RNAs (DNA damage-response RNAs or double-strand break–induced RNAs) required for efficient DNA repair, suggesting Dicer also plays an important role in maintaining genomic stability (*35*, *36*). However, other components of the miRNA/siRNA processing complexes, such as Drosha, DGCR8, or Ago2, have been also implicated in maintaining genomic instability (*37*); nevertheless, ablation of Drosha, DGCR8, or Ago2 does not markedly activate p53 as in Dicer-null cells (*38*–*40*). Thus, our study provides a previously unknown mechanism for Dicer in regulating p53 function independent of its RNA processing activity.

By established Dicer knockout cancer cells, we found that p53 activation occurred in the absence of DNA damage without increasing p53 levels (Fig. 2, A and C), indicating the important role of Dicer in repressing p53 transcriptional activity under normal unstressed conditions. Nevertheless, the levels of p53 activation upon Dicer depletion are not as high as the levels observed under DNA damage conditions, suggesting that increasing p53 levels or additional cofactors are required for fully activation of p53 function. Furthermore, Dicer-mediated repression of p53 function is independent of its intrinsic endoribonuclease activity and does not require other components of the miRNA/siRNA processing complexes, such as Drosha or DGCR8. Instead, by directly interacting with the SUV39H1 histone methyltransferase, Dicer increases the levels of H3K9me3, a histone modification that represses mRNA transcription, on the promoters of p53 target genes. It was reported that SUV39H1 is also involved in Mdm2-mediated transcriptional repression *(**31**)*. However, at the normal unstressed conditions, the levels of Mdm2 are extremely low, whereas the levels of Dicer are relatively high. Thus, it is very likely that the Dicer-SUV39H1 interaction may play a critical role in repressing p53 function in unstressed conditions. The acetylation-dependent regulation of p53 by Dicer is particularly interesting and also raises an important issue whether Dicer is able to recognize other cellular factors in a similar manner. By using acetylated and unacetylated peptides in in vitro binding assays, we found that Dicer is able to interact with an acetylated peptide of FOXO1, but not the unacetylated peptide (fig. S10, A and B). Similar results were also obtained when acetylated and unacetylated peptides of Ku70 (fig. S10C). Thus, it is very likely that Dicer acts as a previously unanticipated reader for acetylation by targeting p53 and potentially other cellular factors. Furthermore, our previous studies showed that the acetylation-defective mutant p53-3KR losses its ability to activate its cellular targets such as p21 and PUMA; however, this mutant retains its ability to activate Mdm2 expression. Dicer suppresses p53-mediated activation of p21 and PUMA but has no obvious effect on Mdm2 (or Tigar) transcription. Upon loss of Dicer expression, the specific transcriptional activity of p53-3KR on p21 and PUMA promoters is largely restored, whereas p53-mediated effect on Mdm2 transcription remains unchanged. Our previous study showed that SET (Protein SET) is also able to regulate p53 function through acetylation on different sites *(**41**).* Although both Dicer and SET are transcriptional repressors for p53 in an acetylation-dependent manner, the molecular mechanisms of the repressions by these two factors are completely different. As an acidic domain–containing protein, SET interacts and represses p53 activity through its binding with the p53 CTD, and SET-mediated effects are dependent on acetylation of the lysine cluster located at the CTD. Moreover, SET represses p53-mediated transactivation by inhibiting p300/CBP-dependent acetylation of H3K18 and H3K27 on p53 target promoters. Last, loss of SET induces activation of almost all the p53 targets including p21, PUMA, and Mdm2. Thus, in contrast to Dicer, SET-mediated repression is not promoter specific. Although future studies are clearly warranted, our data indicate that Dicer is a bona fide transcriptional repressor that governs the expression of p53 target genes through acetylation in a promoter-specific manner.

## MATERIALS AND METHODS

### Cell culture and plasmid generation, transfection, and reagent treatment

Human embryonic kidney–293 (HEK293), H1299 (p53 null), A549 (p53 wild type), HCT116 (p53 wild type), U2OS (p53 wild type), A375 (p53 wild type), MDA-MB-468 (p53 R273H), Cal33 (p53 R175H), and T47D (p53 L194F) cell lines were cultured in Dulbecco’s modified Eagle’s medium (DMEM) or RPMI 1640 supplemented with 10% (v/v) fetal bovine serum (FBS) (Gibco), penicillin (100 U/ml), and streptomycin (100 μg/ml; Gibco). Cells were cultured in a humidified incubator at 37°C and 5% CO_2_. Cal33 cell line was a gift from S. W. Lee. All other cell lines were previously obtained from American Type Culture Collection (ATCC) and have been proven to be negative for mycoplasma contamination. No cell lines used in this work were listed in the International Cell Line Authentication Committee (ICLAC) database. The cell lines were freshly thawed from the purchased seed cells and were cultured for no more than 2 months. The morphology of cell lines was checked every week and compared with the ATCC cell line image to avoid cross-contamination or misuse of cell lines. HCT116 p53-null cell line was generated previously (*41*). MEF cells were cultured in DMEM supplemented with 10% (v/v) 56°C heat-inactivated FBS supplemented with 1% nonessential amino acids. Dicer complementary DNA (cDNA) was purchased from Addgene (plasmid number 19873), and the full-length Dicer or the various fragments were subcloned into pRK5 vectors. The transfection of expressing constructs was performed by Lipofectamine 3000 reagent (Invitrogen, L3000150) according to the manufacturer’s protocol. Doxorubicin-induced DNA damage was performed by 0.2 μg/ml. The proteasome inhibitor MG132 was used at 5 μM for 6 hours. Deacetylase inhibitor trichostatin A (TSA) or nicotinamide (NAM) was used for 6 hours at 1 μM or 10 mM, respectively.

### Complex purification and MS analysis

To generate p53-DBD + TD stable line, its cDNA was cloned into SFB N-terminal vector, and the plasmid was transfected into H1299 cells with Lipofectamine 3000, followed by selection and maintenance with puromycin (1 mg/ml).

For complex purification, the cell pellet was resuspended in Harvest buffer [10 mM Hepes (pH 8.0), 50 mM NaCl, 0.5 M sucrose, 0.1 mM EDTA, and 0.25% Triton X-100] on ice for 5 min. The nuclear pellet was collected after centrifugation and then was resuspended in BC100 buffer [50 mM tris-HCl (pH 7.3), 100 mM NaCl, 0.1 mM EDTA, 0.4% NP-40, and 10% glycerol] on ice for 1 hour. After centrifugation, the supernatant was filtered with 0.45-μm syringe filters and was first immunoprecipitated by Streptavidin beads overnight. The bound peptides were eluted by biotin and were secondly purified by anti–S protein beads overnight. The final elutes by glycine were resolved by SDS–polyacrylamide gel electrophoresis on a 4 to 20% gradient gel and stained by GelCode Blue Stain Reagent (Thermo Fisher Scientific, 24592). Both whole elution and specific visible bands were digested with trypsin and then subjected to LC-MS/MS analysis at Harvard Center for Mass Spectrometry.

### Co-IP assay

Cells were wash with precold phosphate-buffered saline (PBS) twice. Lysate buffer was preadded protease inhibitor cocktail, 1 mM dithiothreitol (DTT) and 1 mM phenylmethylsulfonyl fluoride (PMSF). For total cell lysate, cells were lysed with BC100 buffer on ice for 1 hour and then sonicated for 20 s. Supernatant was collected after centrifuged for 15 min at 15,000 rpm. For cytosolic or nuclear lysate, cells were incubated with Harvest buffer on ice for 5 min for cytosolic fraction. Then, the nuclear pellet was washed twice with 500 μl of buffer A [10 mM Hepes (pH 8.0), 10 mM KCl, 0.1 mM EDTA, and 0.1 mM EGTA], lysed with BC100 buffer, and then sonicated. All lysates were quantified by the Bradford method (Bio-Rad) and further used for IP assay. The indicated antibody (2 μg) was added into lysates and incubated overnight at 4°C, followed by addition of 20 μl of protein A/G agarose for 2 hours. For commercial conjugated beads, cell lysates were directly added 20 μl of Flag M2 Affinity Gel (Sigma-Aldrich, A2220) or Streptavidin beads and incubated overnight at 4°C. After washing with BC100 buffer six times, the interacting components were eluted using 1× loading buffer, glycine, or biotin for further Western blot.

### Whole-cell lysate extraction

Cells were washed twice with precold PBS and lysed with preheated whole-cell lysate buffer [10 mM tris-HCl (pH 8.0), 1% SDS, and 1 mM Na_3_VO_4_] in boiling water for 10 to 20 min. Lysates were sonicated and centrifuged for 10 min at 15,000 rpm to collect supernatant.

### In vitro binding assay

For the in vitro peptide binding assay, HEK293 cells were transfected with indicated Dicer expressed constructs and lysed by Flag lysis buffer [50 mM tris-HCl (pH 8.0), 137 mM NaCl, 1% Triton X-100, 0.2% Sarkosyl, 1 mM NaF, 1 mM Na3VO4, and 10% glycerol] containing protease inhibitor cocktail, 1 mM DTT, and 1 mM PMSF. Lysate were precleared by 20 μl of Streptavidin beads for 4 hours. Then, equal amounts of synthesized biotin-conjugated peptide were incubated with lysates overnight at 4°C, followed by addition of 20 μl of Streptavidin beads for 4 hours the next day. After washing with BC100 buffer six times, the binding components were eluted by boiling with 1× loading buffer and analyzed by Western blot.

For the in vitro pull-down assay, GST or GST-p53 proteins were expressed in bacterial cells and purified with GST Bind Resin (Novagen, 70541). Flag-SUV39H1 proteins were expressed in HEK293 cells and purified with Flag M2 Affinity Gel. Equal amounts of immobilized proteins were incubated with purified Dicer proteins for 4 hours at 4°C, followed by washing with BC100 buffer six times. The binding components were eluted by boiling with 1× loading buffer and analyzed by Western blot.

### Protein purification

The HA-tagged Dicer construct was transfected into HEK293 cells for 24 hours, and the cells were lysed in Flag lysis buffer. HA Affinity Gel was added to supernatant and incubated overnight at 4°C. After washing with Flag lysis buffer six times, the purified proteins were eluted with HA peptide.

### Antibodies

Protein lysates were analyzed by Western blot according to the standard protocols. Information for antibodies is shown: p53 (DO-1; Santa Cruz Biotechnology, sc-126; IP, ChIP, or 1:1000 dilution for Western blot), p53 (CM5, Leica Biosystems; 1:1000 dilution), p53 (FL393; Bioss, bs-8687R; IP or ChIP), p-S15-p53 (Cell Signaling Technology, 9284; 1:1000 dilution), γH2AX (Cell Signaling Technology, 9718; 1:1000 dilution), Mdm2 (Ab-5; Millipore, OP-145; 1:500 dilution), p21 (12D1; Cell Signaling Technology, 2947; 1:1000 dilution), p21 (F-2; Santa Cruz Biotechnology, sc-6246; 1:250 dilution), PUMA (H-136; Santa Cruz Biotechnology, sc-28226; 1:500 dilution), Tigar (E-2; Santa Cruz Biotechnology, sc-166; 1:500 dilution), HA (3F10; Roche, 11867423001; ChIP or 1:2000 dilution for Western blot), myc (9E10; Santa Cruz Biotechnology, sc-40; 1:1000 dilution), Flag (Sigma-Aldrich, F3165; 1:5000 dilution), histone H3 acetyl K18 (Abcam, 1191; ChIP), histone H3 acetyl K27 (Abcam, 4729; ChIP), histone H3 trimethyl K27 (Abcam, 6002; ChIP), histone H3 monomethyl K4 (Abcam, 8895; ChIP), histone H3 trimethyl K9 (Abcam, 8898; ChIP), Vinculin (Sigma-Aldrich, V9131; 1:10,000 dilution), SUV39H1 (Upstate, 05-615; ChIP or 1:500 dilution for Western blot), Dicer (Bethyl, A301-936A; 1:1000 dilution), Dicer (Bethyl, A301-937A; IP), Dicer (Abcam, ab14601; 1:500 dilution), Drosha (Bethyl, A301-886A; 1:1000 dilution), DGCR8 (Bethyl, A302-468A; 1:1000 dilution), Lamin A/C (Bethyl, A303-430A; 1:10,000 dilution), actin (Sigma-Aldrich, A5441; 1:5000 dilution), 53BP1 (Upstate, 05-726; 1:500 dilution), USP28 (Bethyl, A300-898A; 1:2000 dilution), HP1α (Cell Signaling Technology, 2616; ChIP), and p53-lysine 382 acetylated antibody (Cell Signaling Technology, 2525; 1:500 dilution). p53-lysine 120 acetylated antibody (1:500 dilution), p53-lysine 164 acetylated antibody (1:500 dilution), or Sirt1 antibody (1:1000 dilution) was generated by our laboratory. Horseradish peroxidase–conjugated anti-mouse and anti-rabbit secondary antibody (GE Healthcare) and anti-rat (SouthernBiotech) were used.

### Real-time quantitative PCR

Extraction of total RNA was using TRIzol reagent according to the manufacturer’s protocol. For mRNA detection, cDNA was reversed using SuperScript IV VILO Master Mix (Invitrogen) according to the manufacturer’s protocol. mRNA targets’ expression levels were normalized to the expression level of glyceraldehyde-3-phosphate dehydrogenase. Primers used were listed in table S1.

### Chromatin immunoprecipitation–qPCR

Cell were fixed with 1% formaldehyde for 5 to 30 min and stopped by 0.125 M glycine for 10 min. Cells were harvested and lysed by ChIP lysis buffer [10 mM tris-HCl (pH 8.0), 5 mM EDTA,150 mM NaCl, and 0.5% NP-40] for 10 min at 4°C. Cell pellet was spinned down, lysed, and sonicated by radioimmunoprecipitation assay (RIPA) lysis buffer [10 mM tris-HCl (pH 8.0), 5 mM EDTA, 150 mM NaCl, 0.1% SDS, 1% Triton X-100, and 0.5% deoxycholate (DOC)]. The supernatant was collected and precleaned by Protein A salmon sperm DNA agarose (Millipore, 16-157) for 2 hours at 4°C. Equal lysates were incubated with indicated antibody or relative IgG control overnight at 4°C. Next day, 20 μl of Protein A salmon sperm DNA agarose was added into each sample and incubated for 4 hours at 4°C. The agarose was washed with RIPA lysis buffer, high-salt wash buffer [20 mM tris-HCl (pH 8.0), 500 mM NaCl, 5 mM EDTA, 0.1% SDS, and 1% Triton X-100], LiCl wash buffer [10 mM tris-HCl (pH 8.0), 1 mM EDTA, 250 mM LiCl, 1% DOC, and 1% NP-40], and TE buffer [10 mM tris-HCl (pH 8.0) and 1 mM EDTA]. The binding DNA were eluted in elution buffer (1% SDS and 100 mM NaHCO_3_) twice, and reverse cross-linking was performed at 65°C overnight. DNA was extracted using the UltraPure Phenol:Chloroform:Isoamyl Alcohol [25:24:1 (v/v); Invitrogen, 15593] according to the Phenol/Chloroform DNA extraction protocols. Quantitative polymerase chain reaction (qPCR) was performed to detect relative enrichment of each protein or histone modification on targets genes. Primers used were listed in table S2.

### CRISPR-Cas9 system mediated gene ablation

To obtain Dicer, Drosha, or DGCR8 knocking out cell lines, indicated cells were cotransfected with TrueCut Cas9 protein v2 (Invitrogen, A36498) with negative control nontargeting RNA (Invitrogen, A35526) or gene TrueGuide Synthetic guide RNA (Invitrogen, A35533). Forty-eight hours after transfection, cells were diluted and split to single cell for further culture. Knockdown pool cells or single-cell–derived clones were validated by Western blot. The guide RNA ID is shown: for human Dicer: CRISPR762687_SGM and CRISPR762689_SGM; for human DROSHA: CRISPR594074_SGM; for human DGCR8: CRISPR730402_SGM; and for mouse Dicer: CRISPR444003_SGM.

### Luciferase assay

The p21 promoter luciferase assay was performed as previously described. Briefly, in 12-well plate, firefly p21-Luci reporter and *Renilla* control reporter were cotransfected with indicated constructs to H1299 cells for 24 hours, and the relative luciferase activity was measured according to the manufacturer’s protocol of the Dual-Luciferase Reporter Assay System (Promega, E1910) by GloMax Discover Microplate Reader.

### Senescence β-galactosidase staining

Cell senescence was detected by the Senescence β-Galactosidase Staining Kit (Cell Signaling Technology, 9860) according to the manufacturer’s protocol. Briefly, cells were seeded into six-well plate and treated for indicated times. Then, cells were washed with PBS twice, fixed with 1× fixative solution for 15 min at room temperature, and then incubated at 37°C for 16 hours with freshly prepared β-galactosidase staining solution. Images were visualized with microscopy (Olympus IX51).

### Colony formation assay

Five hundred cells were seeded in 6-cm dishes with three biological replicates, treated, and cultured as indicated. Cells were fixed with 4% paraformaldehyde, stained with 0.2% crystal violet solution, and photographed.

### Immunofluorescence for BrdU assay

Cells were seeded and grow on coverslips in six-well plates overnight and then treated with doxorubicin (0.2 μg/ml) for 16 hours, and the cells were pulse-labeled with 10 μM BrdU (BD Biosciences, 550891) for 30 min and then fixed with 4% paraformaldehyde for 30 min at room temperature. After chromosome digesting and blocking, cells were incubated with anti-BrdU (1:500 dilution) overnight at 4°C. Next day, the cells were incubated with the secondary antibodies conjugated with Alexa Fluor 488 (1:1000; Invitrogen, A27023) for 1 hour and then followed by staining with 4′,6-diamidino-2-phenylindole (1:100) for 10 min. Images were visualized with microscopy (Olympus IX51). The percentage of BrdU-positive cells was determined by immunofluorescence.

### Quantification and statistical analysis

A two-tailed unpaired Student *t* test by SPSS 20 was done for the statistical analyses without specific statement. All data represented in the figures with error of mean (means ± SD). *P* < 0.05 was considered statistically significant between groups. Data were graphed using GraphPad Prism 8.0.

## Notes

View/request a protocol for this paper from *Bio-protocol*.
