# Supplementary material for: An unexpected role for Dicer as a reader of the unacetylated DNA binding domain of p53 in transcriptional regulation
Source: Sci Adv. 2021 Oct 27;7(44):eabi6684. doi: 10.1126/sciadv.abi6684 (PMC8550248; doi:10.1126/sciadv.abi6684)
Supplement: Supplementary file 1 — Figs. S1 to S10 Tables S1 and S2 [file sciadv.abi6684_sm.pdf]

Science Advances
AAAS

Supplementary Materials for

An unexpected role for Dicer as a reader of the unacetylated DNA binding domain of p53 in transcriptional regulation

Xin Yang, Xingwu Wang, Zhiming Li, Shoufu Duan, Huan Li, Jian Jin, Zhiguo Zhang, Wei Gu*

*Corresponding author. Email: wg8@cumc.columbia.edu

Published 27 October 2021, *Sci. Adv.* **7**, eabi6684 (2021)
DOI: 10.1126/sciadv abi6684

This PDF file includes:

Figs. S1 to S10
Tables S1 and S2

Figure S1

A

UNIPROT >sp|Q9UPY3|DICER_HUMAN Endoribonuclease Dicer OS=Homo sapiens GN=DICER1 PE=1 SV=3

MKSPALQPLS MAGLQLMTPA SSPMGPFFGL PWQQEAIHDN IYTPRKYQVE LLEAALDHNT IVCLNTGSGK
TFIAVLLTKE LSYQIRGDFS RNGKRTVFLV NSANQVAQQV SAVRTHSDLK VGEYSNLEVN ASWTKERWNQ
EFTKHQVLIM TCYVALNVLK NGYLSLSDIN LLVFDECHLA ILDHPYREIM KLCENCPSCP RILGLTASIL
NGKCDPEELE EKIQKLEKIL KSNAETATDL VVLDRYTSQP CEIVVDCGPF TDRSGLYERL LMELEEALNF
INDCNISVHS KERDSTLISK QILSDCRAVL VVLGPWCADK VAGMMVRELQ KYIKHEQEEL HRKFLLFTDT
FLRKIHALCE EHFSPASLDL KFVTPKVIKL LEILRKYKPY ERQQFESVEW YNNRNQDNYV SWSDSEDDDE
DEEIEEKEKP ETNFPSPFTN ILCGIIFVER RYTAVVLNRL IKEAGKQDPE LAYISSNFIT GHGIGKNQPR
NKQMEAEFRK QEEVLRKFRA HETNLLIATS IVEEGVDIPK CNLVVRFDLP TEYRSYVQSK GRARAPISNY
IMLADTDKIK SFEEDLKTYK AIEKILRNKC SKSVDTGETD IDPVMDDDDV FPPYVLRPDD GGPRVTINTA
IGHINRYCAR LPSDPFTHLA PKCRTRELPD GTFYSTLYLP INSPLRASIV GPPMSCVRLA ERVVALICCE
KLHKIGELDD HLMPVGKETV KYEEELDLHD EEETSVPGRP GSTKRRQCYP KAIPECLRDS YPRPDQPCYL
YVIGMVLTTP LPDELNFRRR KLYPPEDTTR CFGILTAKPI PQIPHFPVYT RSGEVTISIE LKKSGFMLSL
QMLELITRLH QYIFSHILRL EKPALEFKPT DADSAYCVLP LNVVNDSSTL DIDFKFMEDI EKSEARIGIP
STKYTKETPF VFKLEDYQDA VIIPRYRNFD QPHRFYVADV YTDLTPLSKF PSPEYETFAE YYKTKYNLDL
TNLNQPLLDV DHTSSRLNLL TPRHLNQKGK ALPLSSAEKR KAKWESLQNK QILVPELCAI HPIPASLWRK
AVCLPSILYR LHCLLTAEEL RAQTASDAGV GVRSLPADFR YPNLDFGWKK SIDSKSFISI SNSSSAENDN
YCKHSTIVPE NAAHQGANRT SSLENHDQMS VNCRTLLSES PGKLHVEVSA DLTAINGLSY NQNLANGSYD
LANRDFCQGN QLNYYKQEIP VQPTTSYSIQ NLYSYENQPQ PSDECTLLSN KYLDGNANKS TSDGSPVMAV
MPGTTDTIQV LKGRMDSEQS PSIGYSSRTL GPNPGLILQA LTLSNASDGF NLERLEMLGD SFLKHAITTY
LFCTYPDAHE GRLSYMRSKK VSNCNLYRLG KKKGLPSRMV VSIFDPPVNW LPPGYVVNQD KSNTDKWEKD
EMTKDCMLAN GKLDEDYEEE DEEEESLMWR APKEEADYED DFLEYDQEHI RFIDNMLMGS GAFVKKISLS
PFSTTDSAYE WKMPKKSSLG SMPFSSDFED FDYSSWDAMC YLDPSKAVEE DDFVVGFWNP SEENCGVDTG
KQSISYDLHT EQCIADKSIA DCVEALLGCY LTSCGERAAQ LFLCSLGLKV LPVIKRTDRE KALCPTRENF
NSQQKNLSVS CAAASVASSR SSVLKDSEYG CLKIPPRCMF DHPDADKTLN HLISGFENFE KKINYRFKNK
AYLLQAFTHA SYHYNTITDC YQRLEFLGDA ILDYLITKHL YEDPRQHSPG VLTDLRSALV NNTIFASLAV
KYDYHKYFKA VSPELFHVID DFVQFQLEKN EMQGMDSELR RSEEDEEKEE DIEVPKAMGD IFESLAGAIY
MDSGMSLETV WQVYYPMMRP LIEKFSANVP RSPVRELLEM EPETAKFSPA ERTYDGKVRV TVEVVGKGKF
KGVGRSYRIA KSAAARRALR SLKANQPQVP NS

Protein Coverage=559aa/1922aa=29.08%

B

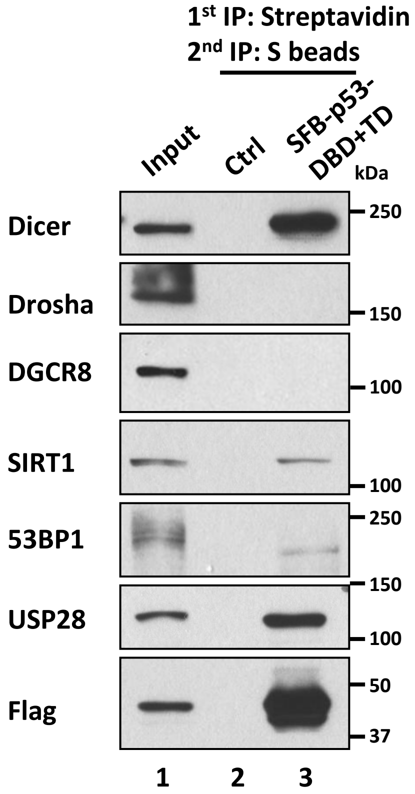

C

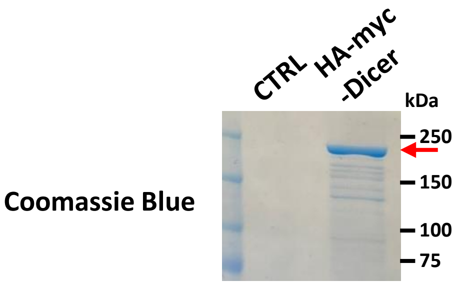

Fig. S1. Dicer identified from p53 associated complexed by liquid chromatography mass spectrometry/mass spectrometry (LC–MS/MS).

(**A**)Total 54 unique Dicer peptides identified from liquid chromatography mass spectrometry/mass spectrometry (LC–MS/MS) are highlighted with 29.08% coverage of the whole Dicer protein. (**B**) Western blot of affinity-purified protein complexes from SFB-p53-DBD+TD stable cell line or the parental H1299 cell line. Data are shown as representative of three experiments. (**C**) Coomassie blue staining and western blots of purified HA-myc tagged Dicer full-length protein.

Figure S2

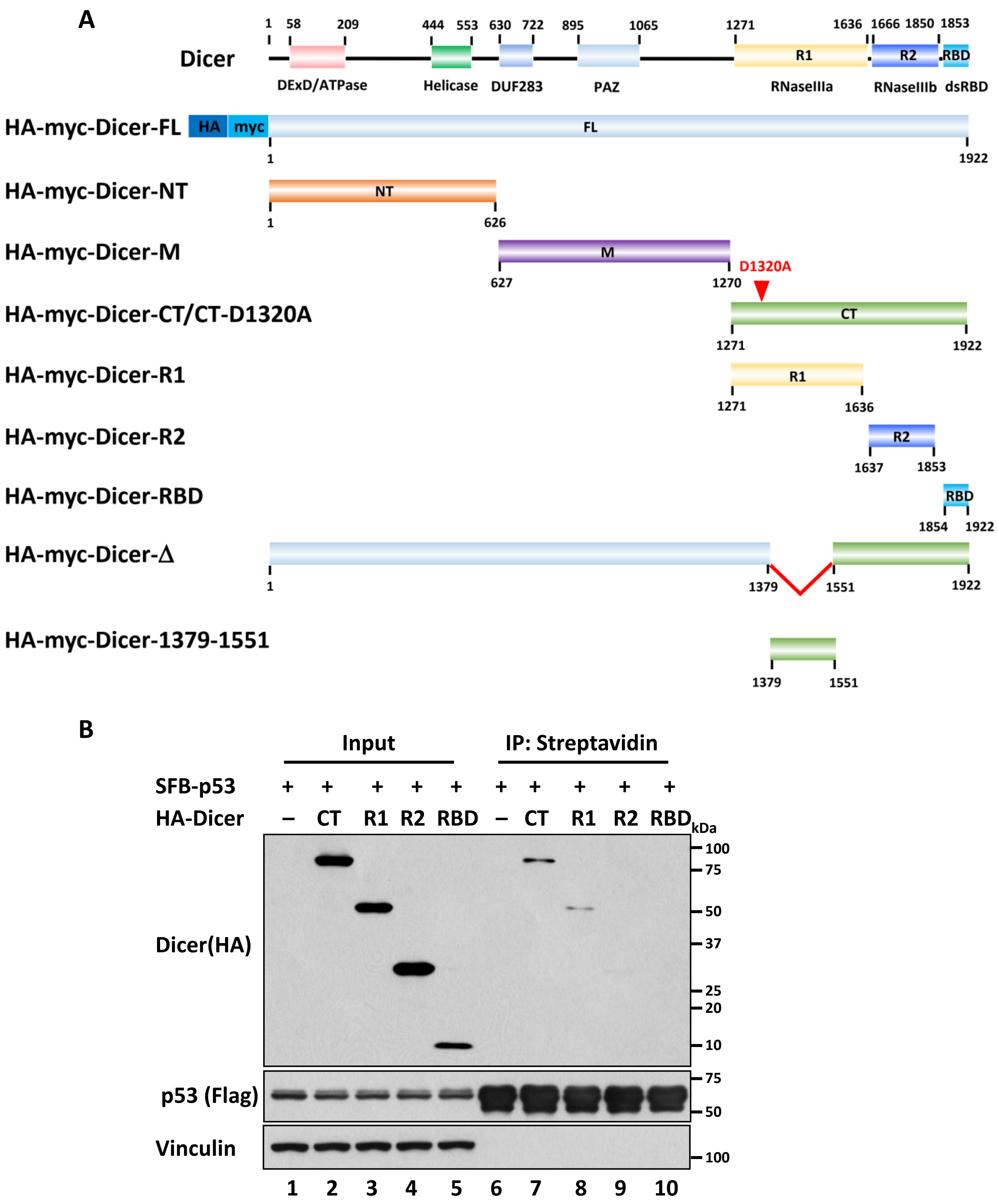

Fig. S2. Subdomain mapping of the p53–Dicer interaction.

(**A**) Schematic diagram of the Dicer domain and Dicer isoform used in this study. R1, RNase IIIa domain, aa. 1271-1636; R2, RNase IIIb domain, aa. 1637-1853; RBD, dsRBD, double-stranded RNA binding domain, aa. 1854-1922. (**B**) Western blot analysis of p53 and Dicer domains for their interaction. Data are shown as representative of three experiments.

Figure S3

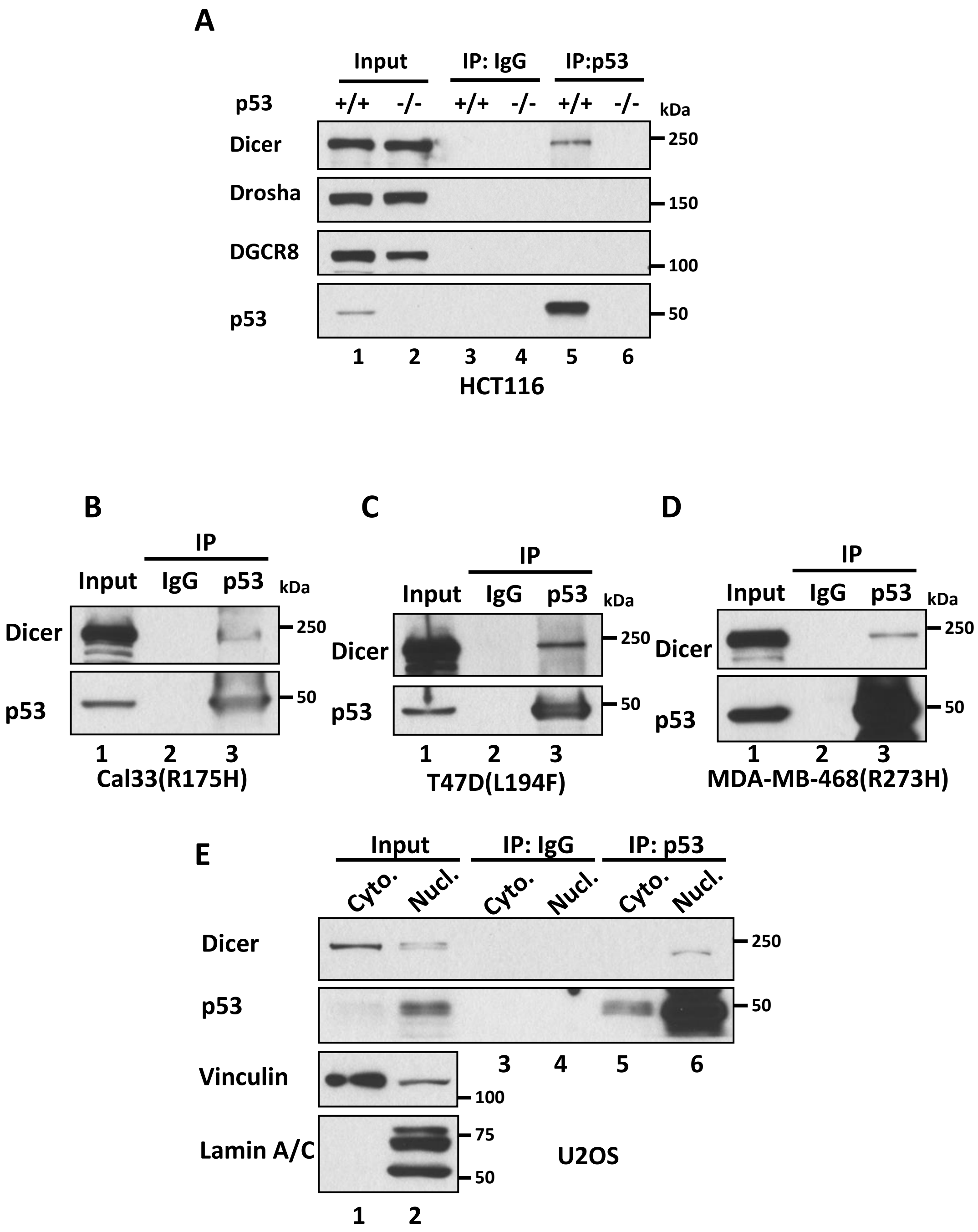

Fig. S3. Further analysis of the p53–Dicer interaction.

(**A**) Western blot analysis of endogenous interaction among p53, Dicer, Drosha, and DGCR8 in HCT116 p53 wildtype or null cells. (**B-D**) Western blot analysis of endogenous interaction among p53 and Dicer in Cal33 p53 R175H cells (**B**), T47D p53 L194F cells (**C**), or MDA-MB-468 p53 R273H cells (**D**). (**E**) Western blot analysis of the interaction between p53 and Dicer in the cytoplasm (Cyto.) or nucleus (Nucl.) fraction of U2OS cells upon MG132 treatment. All data are shown as representative of three experiments.

Figure S4

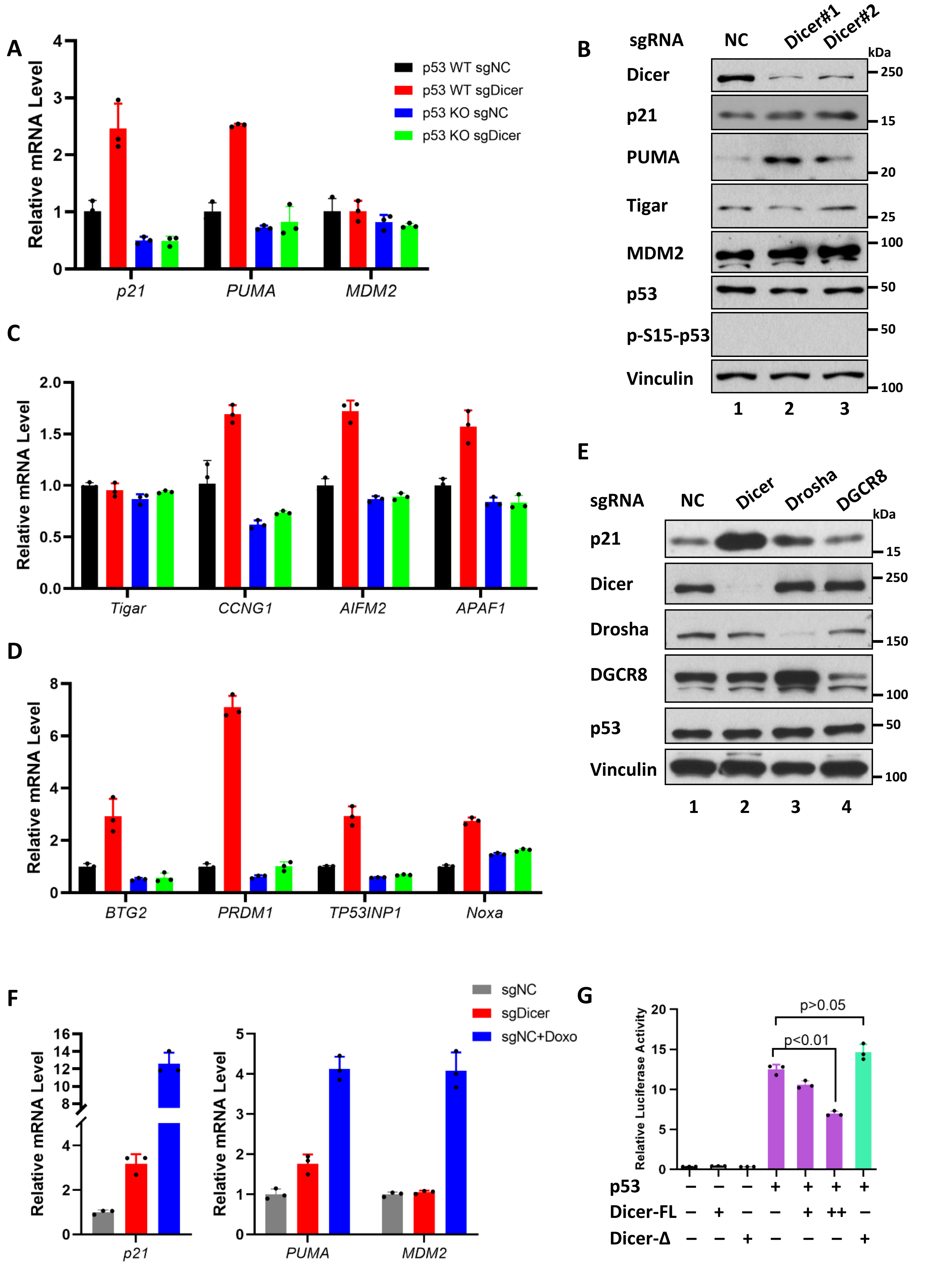
A
Relative mRNA Level
p53 WT sgNC
p53 WT sgDicer
p53 KO sgNC
p53 KO sgDicer
p21
PUMA
MDM2
B
sgRNA
NC
Dicer#1
Dicer#2
kDa
Dicer
p21
PUMA
Tigar
MDM2
p53
p-S15-p53
Vinculin
250
15
20
25
100
50
50
100
1
2
3
C
Relative mRNA Level
Tigar
CCNG1
AIFM2
APAF1
D
Relative mRNA Level
BTG2
PRDM1
TP53INP1
Noxa
E
sgRNA
NC
Dicer
Drosha
DGCR8
kDa
p21
Dicer
Drosha
DGCR8
p53
Vinculin
15
250
150
100
50
100
1
2
3
4
F
sgNC
sgDicer
sgNC+Doxo
Relative mRNA Level
p21
PUMA
MDM2
G
Relative Luciferase Activity
p>0.05
p<0.01
p53
Dicer-FL
Dicer-Δ

Fig. S4. Specific effects of Dicer in regulating p53 function.

(**A, C, D**) qPCR assay of the p53 targets regulated by the p53 and/or Dicer knockout in HCT116 cells. Error bars indicate mean$\pm$SD, n=3 technical replicates. (**B**) Western blot analysis of the effect of Dicer CRISPR-Cas9 knockout clones on p53 activity in A549 cells. (**E**) Western blot analysis of the effect of Dicer, Drosha, or DGCR8 Crispr-Cas9 knockdown on p53 activity in HCT116 cells. (**F**) qPCR assay of the *p21*, *PUMA* and *MDM2* genes regulated by the Dicer knockout or Doxorubicin treatment in HCT116 cells. Error bars indicate mean$\pm$SD, n=3 technical replicates. (**G**) Luciferase assays of Dicer-mediated regulation of p53 transcriptional activity in H1299 cells by transfected p53 or p53 plus Dicer or Dicer-Δ. Error bars indicate mean$\pm$SD, n=3 biological replicates. All data are shown as representative of three experiments.

Figure S5

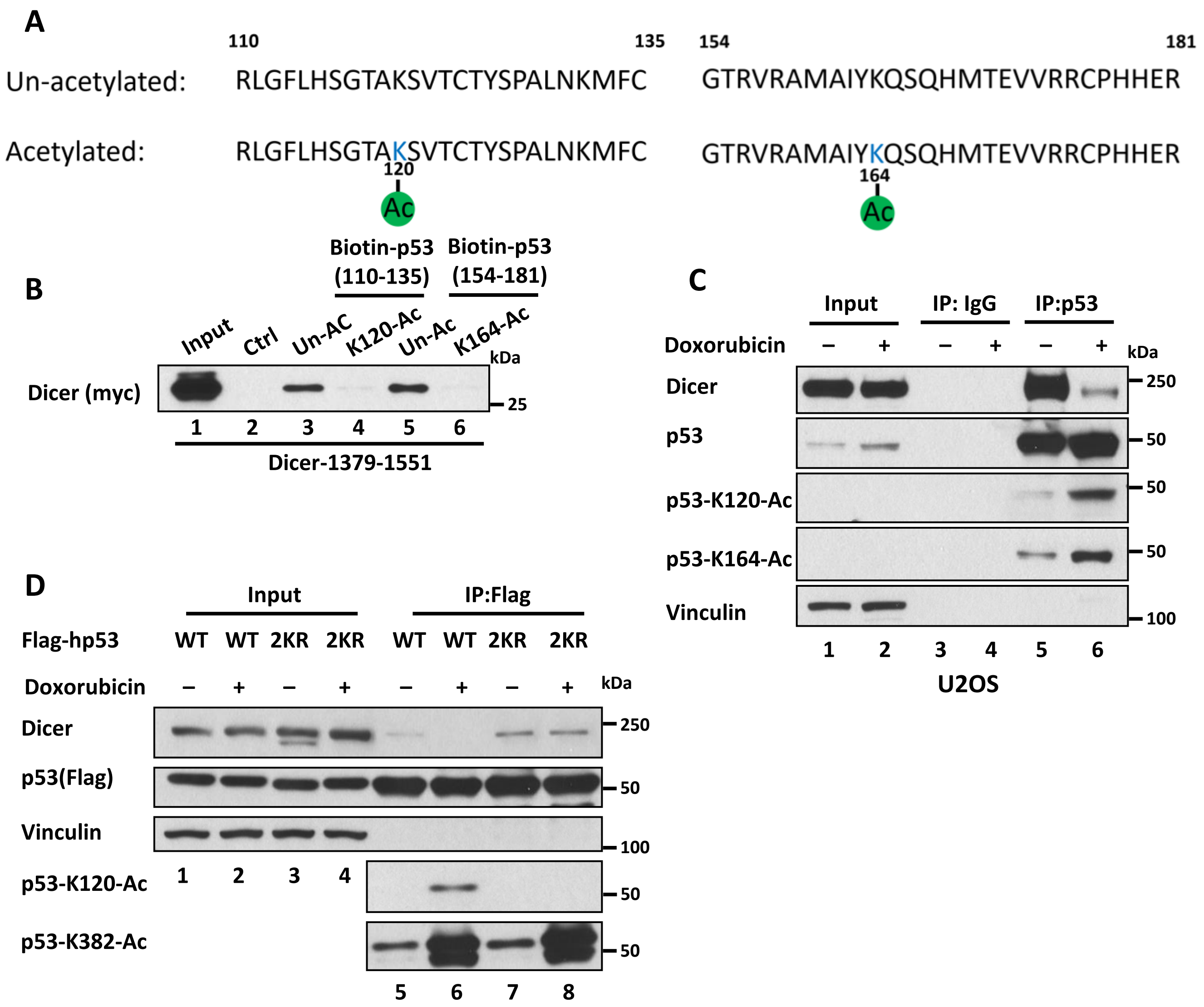

Fig. S5 Regulation of the Dicer-p53 interaction by acetylation.

(**A**) Sequence of the acetylated or unacetylated peptides of p53-(aa. 110-135)-K120 and p53 (aa. 154-181)-K164. (**B**) *In vitro* binding assay of biotin-conjugated K120 and K164 unacetylated or acetylated p53 and Dicer-1379-1551. (**C**) Western blot analysis of the endogenous interaction between p53 and Dicer upon MG132 plus TSA/NAM treatment or Doxorubicin (0.2 µg/ml) plus TSA/NAM treatment of U2OS cells. (**D**) Western blot analysis of the interaction between p53 wildtype and 2KR (K120R, K164R) and Dicer upon DNA damage treatment in H1299 cells. All data are shown as representative of three experiments.

Figure S6

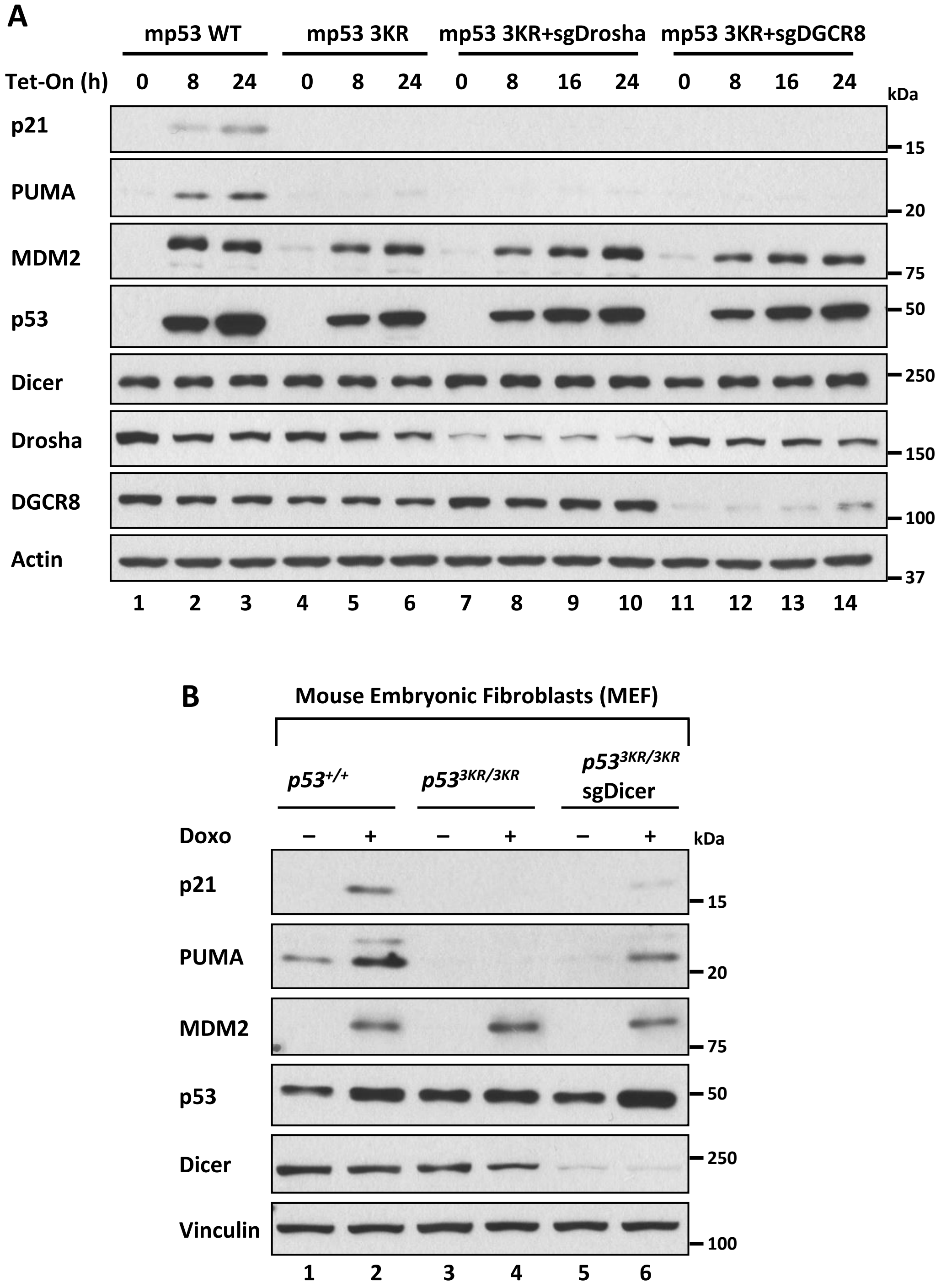

Fig. S6 Dicer, but not Drosha or DGCR8, is involved in p53 acetylation mediated transactivation.

(**A**) Western blot analysis of p53 activity in H1299 mouse p53 WT, 3KR, 3KR Drosha knockdown, or 3KR DGCR8 knockdown Tet-On cells with or without induction in time dependent manner. (**B**) Western blot analysis of p53 activity in p53 wildtype MEF, *p53*$^{3KR/3KR}$ MEF or *p53*$^{3KR/3KR}$ MEF by Dicer knockdown with or without Doxorubicin treatment. All data are shown as representative of three experiments.

Figure S7

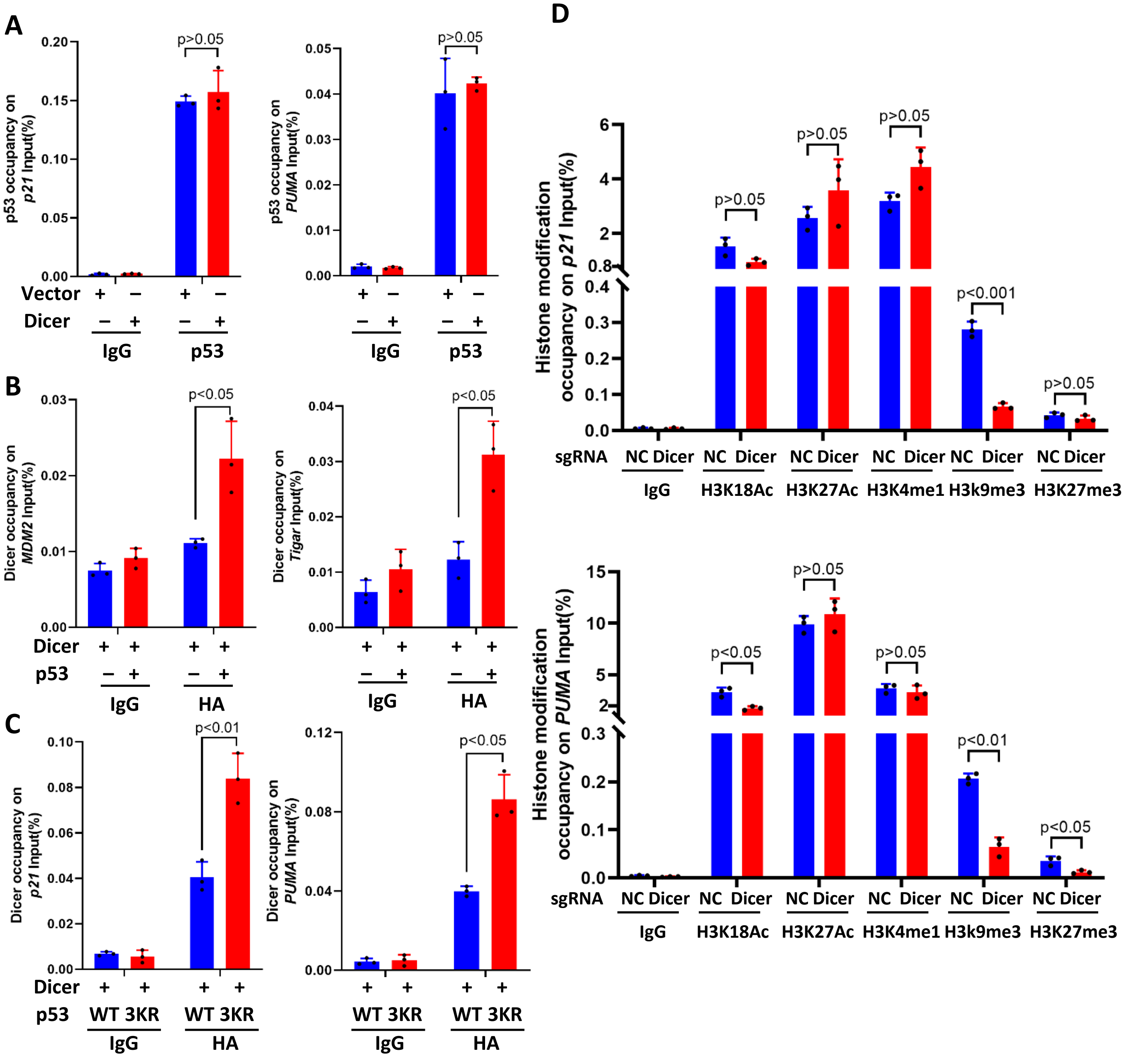

Fig. S7. Mechanistic studies of Dicer-mediated regulation of p53 function.

(**A**) ChIP analysis of the Dicer knockout mediated effect on p53 at the *p21* and *PUMA* promoter in HCT116 cells. (**B**) ChIP analysis of the p53 effect on Dicer at the *MDM2* or *Tigar* promoter in H1299 cells by transfected Dicer with or without p53. (**C**) ChIP analysis of the p53 acetylation effect on Dicer at the *p21* and *PUMA* promoter in H1299 by transfected Dicer with mouse p53 wildtype or 3KR. (**D**) ChIP analysis of the Dicer knockout mediated effect on histone modifications (H3K18Ac, H3K27Ac, H3K4me1, H3K9me3, and H3K27me3) at the *p21* and *PUMA* promoter in HCT116 cells. All data are shown as representative of at least three experiments. Error bars indicate mean±SD, n=3 technical replicates.

Figure S8

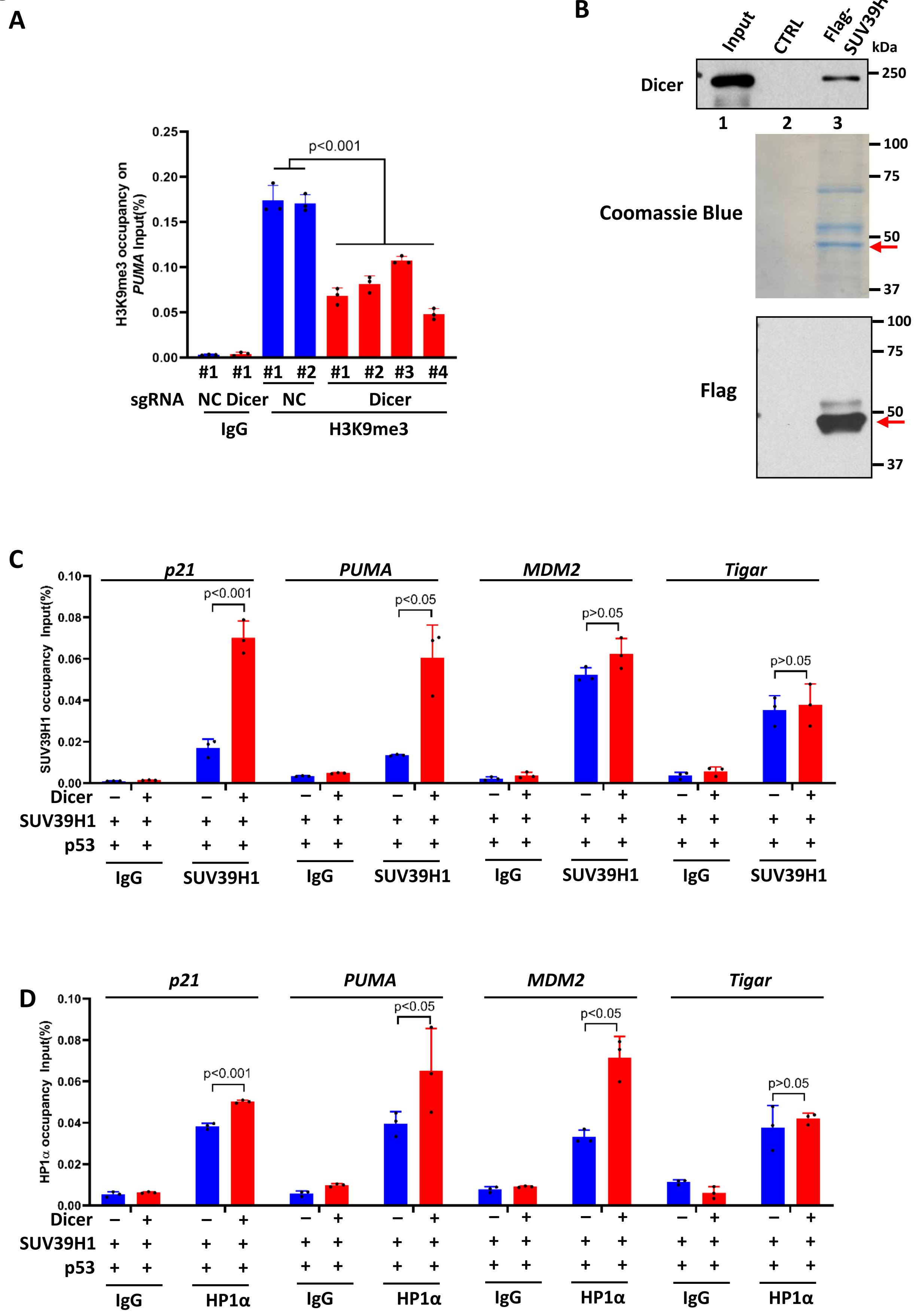
A
H3K9me3 occupancy on PUMA Input(%)
0.25
0.20
0.15
0.10
0.05
0.00
p<0.001
#1 #1 #1 #2 #1 #2 #3 #4
sgRNA
NC Dicer NC Dicer
IgG
H3K9me3
B
Input
CTRL
Flag-SUV39H1
kDa
Dicer
250
1 2 3
100
75
Coomassie Blue
50
37
100
75
Flag
50
37
C
p21
PUMA
MDM2
Tigar
SUV39H1 occupancy Input(%)
0.10
0.08
0.06
0.04
0.02
0.00
p<0.001
p<0.05
p>0.05
p>0.05
Dicer
SUV39H1
p53
IgG
SUV39H1
IgG
SUV39H1
IgG
SUV39H1
IgG
SUV39H1
D
p21
PUMA
MDM2
Tigar
HP1α occupancy Input(%)
0.10
0.08
0.06
0.04
0.02
0.00
p<0.001
p<0.05
p<0.05
p>0.05
Dicer
SUV39H1
p53
IgG
HP1α
IgG
HP1α
IgG
HP1α
IgG
HP1α

Fig. S8. Dicer-mediated promoter specific regulation of p53 function is dependent on SUV39H1.

(**A**) ChIP analysis of the Dicer knockout mediated effect on H3K9 tri-methylation at the *PUMA* promoter region in HCT116 cells. Two negative control clones and four Dicer knockout clones were examined. Significance was assessed in indicated comparisons by One-way analysis of variance (ANOVA). (**B**) *In vitro* binding assay of Flag-SUV39H1 and purified Dicer. (**C**) ChIP analysis of the Dicer mediated effect on SUV39H1 at the *p21*,*PUMA*, *MDM2*, and *Tigar* promoter in H1299 cells. (**D**) ChIP analysis of the Dicer mediated effect on HP1α at the *p21*,*PUMA*, *MDM2*, and *Tigar* promoter in H1299 cells. All data are shown as representative of at least three experiments. Error bars indicate mean±SD, n=3 technical replicates.

Figure S9

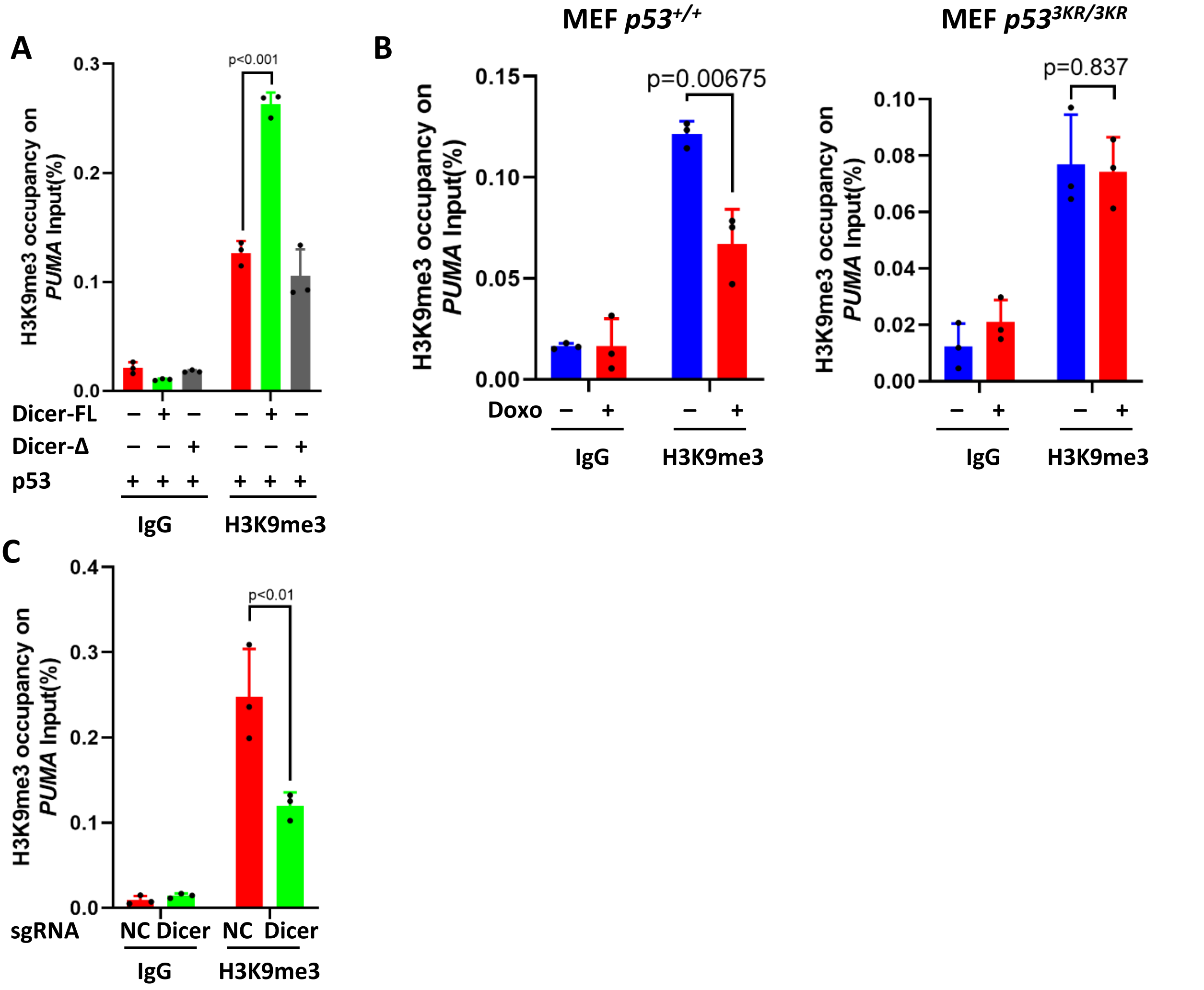

Fig. S9. Dicer regulates the levels of H3K9 tri-methylation of p53 target promoters.

(**A**) ChIP analysis of overexpressed Dicer effect on H3K9 tri-methylation at the *PUMA* promoter in H1299 by transfected p53 or p53 plus FL Dicer or Deficit Dicer. (**B**) ChIP analysis of the H3K9 tri-methylation at the *PUMA* promoter in MEF $p53^{+/+}$ (Left part) or MEF $p53^{3KR/3KR}$ (Right part) cells with TSA/NAM or Doxorubicin (Doxo, 0.2 µg/ml) plus TSA/NAM treatment. (**C**) ChIP analysis of the Dicer effect on H3K9 tri-methylation at the *PUMA* promoter in H1299 mouse p53 3KR tet-on sgNC or sgDicer cells with doxycycline (0.1µg/mL). All data are shown as representative of at least three experiments. Error bars indicate mean±SD, n=3 technical replicates.

Figure S10

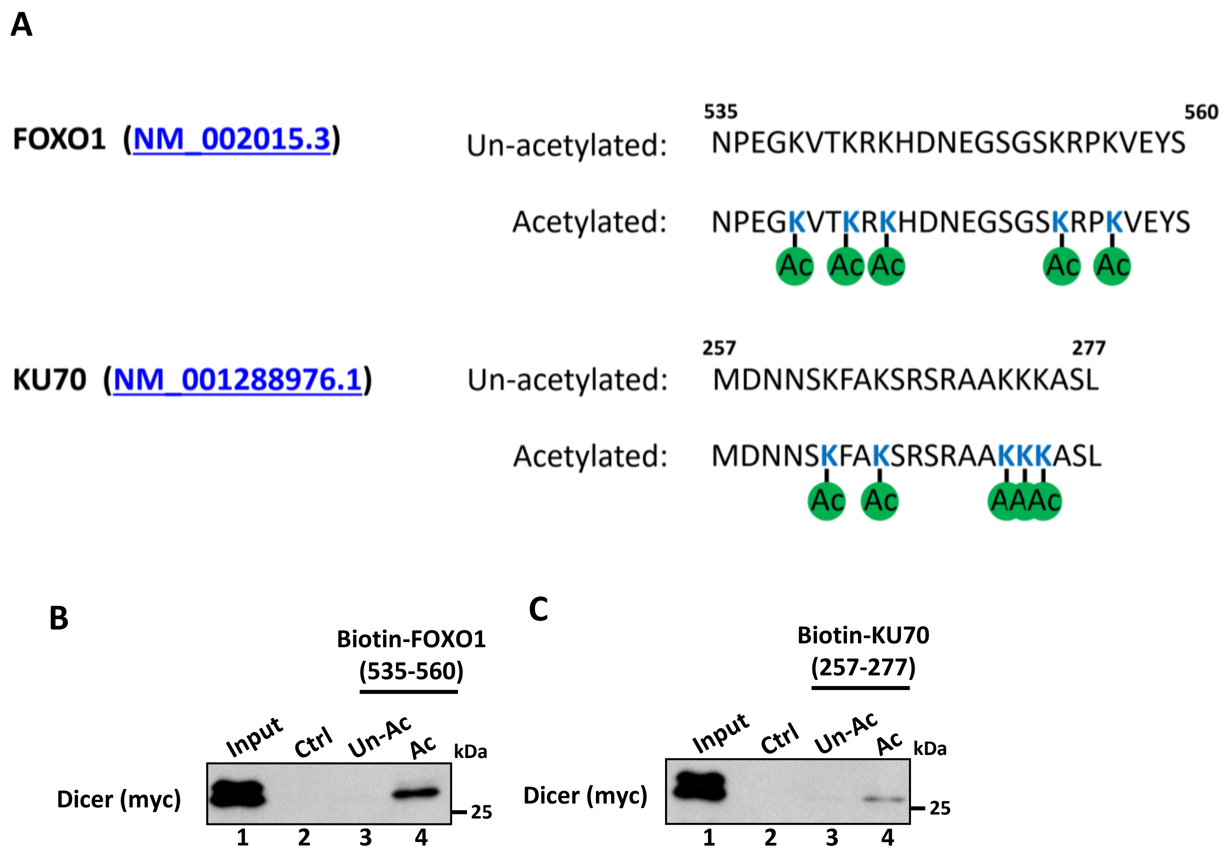

Fig. S10. Regulation of the interactions between Dicer and other cellular factors by acetylation.

(**A**) Sequence of the acetylated or unacetylated peptides of FOXO1 (aa. 535-560) and KU70 (aa. 257-277). (**B** and **C**) *In vitro* binding assay of unacetylated or acetylated FOXO1 or KU70 and Dicer-1379-1551. Data are shown as representative of three experiments.

Table S1. Oligos used for RT-qPCR assay in this study

Primer Name	Sequence(5’-3’)
p21 F	CTGTCACTGTCTTGTACCCTTGT
p21 R	GGTAGAAATCTGTCATGCTGGT
PUMA F	GACCTCAACGCACAGTACGAG
PUMA R	AGGAGTCCCATGATGAGATTGT
MDM2 F	CATTCAGGTGATTGGTTGGA
MDM2 R	CACAGTAACTTGATATACCTC
Tigar F	GACTTCGGGAAAGGAAATACG
Tigar R	CACTCTTCCCTGGCTGCTTTG
CCNG1 F	GCTGTGAATTTACTGGACAG
CCNG1 R	TCGGATCAAGTCAGTTGCC
AIFM2 F	ACATGAAGGACTCCTTCCAC
AIFM2 R	CCTTGAAAGTCACCGAGTAAG
APAF1 F	AACCAGGATGGGTCACCATA
APAF1 R	ACTGAAACCCAATGCACTCC
BTG2 F	AGCGAGCAGAGGCTTAAGGT
BTG2 R	GATGATGGGGTCCATCTTGT
PRDM1 F	GACCGGCTACAAGACCCTTCCCTAC
PRDM1 R	ATGTGGCTTTTCTCCCGTGTGTACC
TP53INP1 F	GTGAAGTCAGTTCTTCCTC
TP53INP1 R	CTGCTGAGAAACCAGTGCAAG
NOXA F	GCAGAGCTGGAAGTCGAGTGT
NOXA R	CTCTTTTGAAGGAGTCCCCTCAT
GAPDH F	GAAGGTGAAGGTCGGAGTC
GAPDH R	GAAGATGGTGATGGGATTTC

Table S2. Oligos used for ChIP-qPCR assay in this study

Primer Name	Sequence(5’-3’)
Human p21 F	TGATTGGCTTTCTGGCCGTC
Human p21 R	CCAGCCTCTTCTATGCCAGAGC
Human PUMA F	CGGAATGGAAAGCTATGAGACA
Human PUMA R	CACAAATCTGGCAGGGGAC
Human MDM2 F	GTCAAGTTCAGACACGTTC
Human MDM2 R	CCTCCAATCGCCACTGAACAC
Human Tigar F	CGGCAGGTCTTAGATAGCTT
Human Tigar R	GGCAGCCGGCATCAAAAACA
Mouse p21 F	CAGGCCTGGGTCTGTTCA
Mouse p21 R	TCTCCACCACCCTGCACT
Mouse PUMA F	GAGGAAGGGTCACTGTTTC
Mouse PUMA R	GACAAATGGGGAAGAGGC
